# Astrocytic noncanonical WNT5B signaling modulates extracellular matrix remodeling and neuropathology in Huntington’s disease

**DOI:** 10.1038/s41392-025-02545-9

**Published:** 2026-01-19

**Authors:** Phuong Thi Thanh Nguyen, Ali Yousefian-Jazi, Seung Jae Hyeon, Soomin Lee, Seung Chan Kim, Uiyeol Park, Yeeun Jeong, Sojung Kim, Suhyun Kim, Yeyun Kim, Hannah L. Ryu, Kyung Eun Lee, Thor D. Stein, Richard H. Myers, Eun Mi Hwang, Junghee Lee, Hoon Ryu

**Affiliations:** 1 Center for Brain Disorders, Brain Science Institute, Korea Institute of Science and Technology (KIST), Seoul, Republic of Korea; 2 KIST School, Division of Bio-Medical Science & Technology, University of Science and Technology, Seoul, Republic of Korea; 3 Department of Integrated Biosystem and Biomedical Sciences, Korea University, Seoul, Republic of Korea; 4 Department of Medicine, Hanyang University Medical School, Seoul, Republic of Korea; 5 Department of Integrated Biomedical and Life Science, College of Health Science, Korea University, Seoul, Republic of Korea; 6 Boston University Alzheimer’s Disease Center and Department of Neurology, Boston University Chobanian & Avedisian School of Medicine, Boston, MA USA; 7 Biochemistry Program, Wellesley College, Wellesley, MA USA; 8 Advanced Analysis Data Center, KIST, Seoul, Republic of Korea; 9 Neurology Research Group, VA Boston Healthcare System, Boston, MA USA; 10 Genome Science Institute, Boston University Chobanian and Avedisian School of Medicine, Boston, MA USA; 11 KHU-KIST Department of Converging Science and Technology, Kyung Hee University, Seoul, Republic of Korea; 12 Present Address: Weill Institute for Neurosciences, Department of Neurology, University of California, San Francisco, San Francisco, CA USA

**Keywords:** Epigenetics, Neurological disorders, Molecular neuroscience

## Abstract

Huntington’s disease (HD) is a fatal neurodegenerative disorder characterized by a triad of behavioral symptoms: involuntary movement, emotional change, and cognitive dysfunction. Although alterations in WNT signaling have been reported in HD, its precise role in pathogenesis remains unclear. In this study, we found that astrocytic *WNT5B* mRNA and protein levels are elevated in the striatum of both HD patients and HD model mice. The noncanonical WNT5B signaling pathway induced sustained expression of matrix metallopeptidase 14 (*MMP14*), an extracellular matrix (ECM)-degrading enzyme, via activation of the NFATc2 transcription factor in both human and primary mouse astrocytes. Robust upregulation of MMP14 led to ECM degradation, medium spiny neuron (MSN) damage, and increased mutant huntingtin aggregation in N171-82Q HD transgenic mice. Furthermore, *WNT5B* gain-of-function exacerbated neuropathology, impaired motor coordination, and shortened the lifespan of N171-82Q mice. We further demonstrated that the overexpression of the estrogen receptor *α* (ER*α*) suppresses NFATc2 transcriptional activity in vitro. A targeted therapy for the WNT5B-NFATc2-MMP14 signaling pathway by genistein, a phytoestrogen, reduced *MMP14* transcription by antagonizing NFATc2 activity and preventing ECM degradation in N171-82Q mice. Genistein treatment also ameliorated neuropathology and motor deficits and prolonged the lifespan of HD mice. Together, these findings define a molecular pathological mechanism in which astrocytic *MMP14* transcription, driven by the noncanonical WNT5B signaling pathway, promotes ECM degradation and MSN damage and accelerates neurodegeneration in HD. Modulation of the noncell-autonomous WNT5B-NFATc2-MMP14 signaling pathway by genistein may serve as a potential therapeutic strategy for mitigating HD pathogenesis.

## Introduction

Huntington’s disease (HD) is a devastating, autosomal-dominant neurodegenerative disorder characterized by a progressive triad of motor dysfunction, cognitive decline, and psychiatric disturbances. HD arises from an abnormal expansion of the CAG trinucleotide repeat in the Huntingtin (HTT) gene, which encodes a mutant HTT (mHTT) protein containing an expanded polyglutamine (polyQ) tract. This expanded polyQ domain confers toxic gain-of-function properties that promote aberrant protein folding and aggregation, leading to the formation of intracellular inclusion bodies and proteolytic fragments that disrupt numerous essential cellular processes. Among the best-characterized mechanisms is the ability of mHTT to abnormally bind transcription factors and chromatin-modifying enzymes, thereby inducing widespread transcriptional dysregulation and profound epigenetic reprogramming in vulnerable neuronal populations.^1–4^ Although neurons, particularly striatal medium spiny neurons (MSNs) are most susceptible, mHTT aggregates are also present in all major glial cell types, including astrocytes, microglia, and oligodendrocytes.^5^ The functional consequences of glial mHTT accumulation have gained increasing attention, as growing evidence indicates that glial pathology is not merely a secondary response but an active contributor to neurodegeneration. For example, astrocyte-specific expression of mHTT has been shown to impair glutamate uptake, calcium buffering, and synaptic support, leading to MSN vulnerability and behavioral deficits in HD mouse models.^6^ These findings highlight the importance of understanding astrocytic pathogenic mechanisms and raise the possibility that glial reprogramming could slow disease progression.

Dysregulation of the wingless-type MMTV integration site family (WNT) signaling pathway has emerged as a prominent feature of multiple neurodegenerative conditions, including Parkinson’s disease (PD) and HD, where synaptic dysfunction represents an early hallmark of pathology.^7–9^ The WNT pathway encompasses canonical (β-catenin–dependent) and noncanonical branches, each of which regulates distinct aspects of neural development and adult brain homeostasis. Canonical WNT–β-catenin signaling governs stem cell proliferation, neurogenesis, and transcriptional regulation, while the noncanonical WNT–Ca²⁺ and WNT–planar cell polarity (PCP) pathways control cytoskeletal organization, cell polarity, migration, and calcium dynamics. Activation of the noncanonical WNT/Ca²⁺ arm stimulates the calcium-responsive nuclear factor of activated T cells (NFAT) transcription factors, enabling finely tuned regulation of gene expression programs.^10,11^ mHTT perturbs this system by interfering with β-catenin turnover through direct interactions with components of the β-catenin destruction complex, thereby promoting excessive stabilization of β-catenin and altering downstream transcription.^12^ Given that both canonical and noncanonical WNT pathways influence neuronal–glial communication, synaptic remodeling, and inflammatory responses, delineating how mHTT alters WNT signaling in specific cell types is essential for understanding the full spectrum of HD pathogenesis.

Pharmacological modulation of WNT signaling has garnered considerable interest as a potential therapeutic avenue for neurodegenerative diseases. Multiple WNT-targeting compounds are under development for Alzheimer’s disease (AD), PD, and amyotrophic lateral sclerosis (ALS), reflecting the pathway’s central role in neuronal survival, inflammation, and synaptic integrity.^13^ However, to date, very few WNT modulators have been investigated in HD models; one exception is indomethacin, a nonsteroidal anti-inflammatory drug capable of reducing cellular β-catenin levels and attenuating polyQ-HTT–induced neuronal toxicity in primary striatal cultures expressing either wild-type HTT (480-17Q) or mutant HTT (480-68Q).^14^ Emerging evidence suggests that dysregulated WNT signaling in astrocytes may be particularly consequential, contributing to both reactive and dysfunctional states that compromise synaptic homeostasis, metabolic support, and neuroimmune regulation.^15,16^ Moreover, the interplay between WNT signaling and extracellular matrix (ECM) remodeling has recently been recognized as a critical determinant of neural circuit stability and disease progression.^17^ Within this framework, identifying astrocyte-specific WNT mechanisms that regulate ECM dynamics could reveal molecular targets capable of modifying disease trajectory. Genistein, a naturally occurring isoflavone derived from soybeans, has gained attention for its anti-inflammatory, antitumor, and antiangiogenic properties, coupled with an excellent safety profile even at high doses.^18,19^ Its pleiotropic biological activities have prompted exploration of its therapeutic potential in several neurodegenerative disorders.^20^ Nevertheless, the specific molecular pathways through which genistein acts in the central nervous system—particularly in relation to WNT signaling and glial pathology in HD—remain largely unexplored.

In this study, we investigated the role of astrocytic WNT signaling in the pathogenesis of HD and sought to determine whether genistein modulates WNT activity to exert neuroprotective effects. Our analyses reveal that WNT5B expression is robustly elevated in astrocytes derived from both HD patients and HD mouse models. Mechanistic studies further show that WNT5B activates the noncanonical WNT signaling cascade in an NFATc2-dependent manner in striatal astrocytes, both in vitro and in vivo. Activation of astrocytic WNT5B triggers ECM degradation through the NFATc2–MMP14 axis, contributing to neuropathological alterations characteristic of HD. Finally, we demonstrate that genistein modulates this pathway by attenuating NFATc2-driven induction of MMP14, thereby reducing ECM breakdown and ameliorating neuropathological and behavioral deficits in HD mice. Collectively, these findings identify astrocytic WNT5B–NFATc2–MMP14 signaling as a critical pathological mechanism in HD and establish genistein as a promising modulator of astrocytic dysfunction with therapeutic potential.

## Results

### WNT5B is upregulated in the striatal astrocytes of HD postmortem brains and HD mouse models

**Fig. 1:**
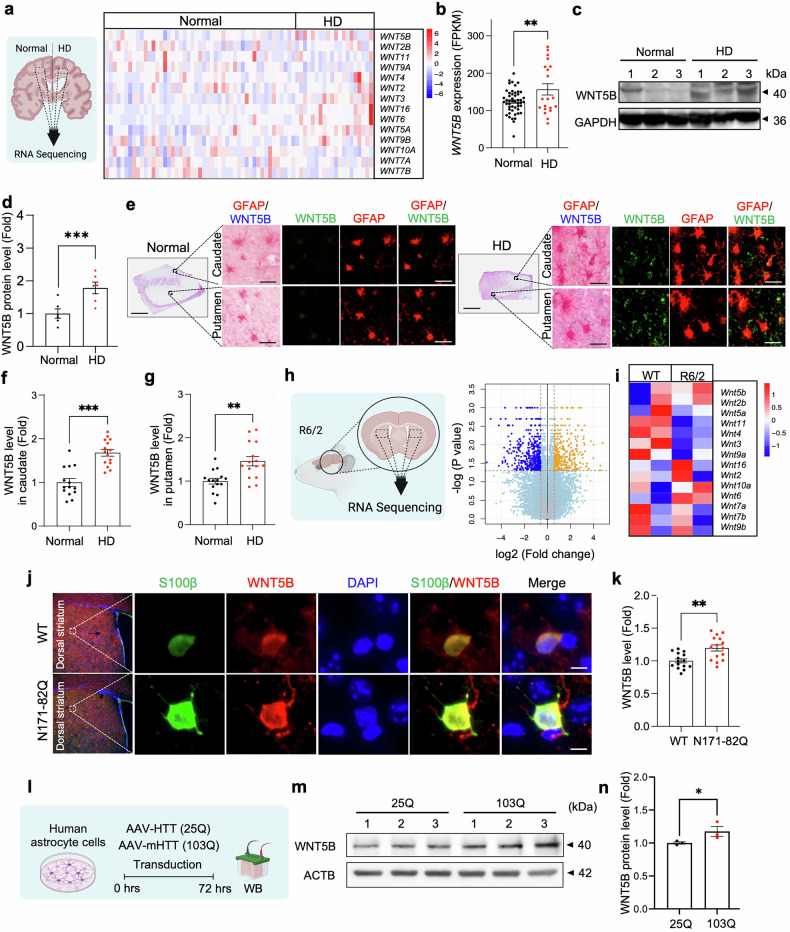
WNT5B is upregulated in the striatal astrocytes of HD patients and HD model mice. (**a**) Heatmap showing the expression levels of WNT signaling-related genes in normal subjects (Normal) and HD patients (HD). Data were extracted from GSE64810.^21^ **(b)** Expression levels of WNT5B in the prefrontal cortex of 20 HD patients and 49 neuropathologically normal subjects. The error bars represent the means ± SEMs. Student’s t test was used (**, *p* < 0.01). **(c)** Western blot analysis of WNT5B protein levels in postmortem striatal tissues from normal and HD patients. **(d)** Densitometry analysis of WNT5B protein levels (*n* = 5 patie*n*ts per group: normal subjects and HD patients). Student’s t test was performed (***, *p* < 0.001). **(e)** Double chromogenic immunostaining images for WNT5B (blue) and GFAP (red) in the striatum of normal and HD patients. Scale bars (black): top, 2 mm; bottom, 10 μm. **(f)** Quantification of WNT5B immunoreactivity in GFAP-positive cells in the caudate. A total of 15 cells per group were analyzed (5 cells per case, *N* = 3 cases per group: normal subjects and HD patients). Significantly different at ***, *p* < 0.001. **(g)** Quantification of WNT5B immunoreactivity in GFAP-positive cells in the putamen. A total of 15 cells per group were analyzed (5 cells per case, *N* = 3 cases per group). Significant differences at **, *p* < 0.01. **(h)** Volcano plot showing differentially expressed genes in R6/2 HD transgenic mice (*n* = 2) compared with WT mice (*n* = 2). **(i)** Heatmap showing the expression levels of WNT signaling-related genes in WT and R6/2 transgenic mice. **(j)** Immunostaining of WNT5B and S100β in WT and N171-82Q mice. Nuclei were counterstained with DAPI. Scale bars (white): 10 μm. **(k)** Quantitative analysis of WNT5B immunoreactivity in S100β-positive cells. A total of 15 cells per group were analyzed (5 cells per mouse; *N* = 3 mice per group: WT and N171-82Q). Significant differences at **, *p* < 0.01. **(l)** Experimental design for Western blot analysis of human astrocytes transduced with wild-type *HTT* (wt*HTT*) (25Q) or mutant *HTT* (m*HTT*) (103Q) for 72 h. **(m)** Western blot analysis of WNT5B protein levels in human astrocytes transfected with wt*HTT* (25Q) or m*HTT* (103Q). **(n)** Densitometry analysis of WNT5B protein levels (3 samples per group: 25Q and 103Q). Significantly different at *, *p* < 0.05

To investigate how WNT signaling contributes to HD pathologies, we performed RNA sequencing (RNA-seq) on the prefrontal cortex of 20 HD patients and 49 neuropathologically normal controls.^21^ Among the 19 WNT genes examined, WNT5B presented the highest basal expression and was markedly upregulated in HD patients (Fig. 1a, b and Supplementary Fig. 1a). While *WNT6* was also increased in the transcriptomic dataset, its relatively low basal expression level compared with that of *WNT5B* in both the human brain and human astrocytes (Supplementary Fig. 1b) indicates that *WNT5B* is the more functionally relevant isoform in the context of HD pathogenesis. Interestingly, the increase in WNT5B expression was not significantly correlated with CAG repeat length, disease duration, or disease grade in the HD patient cohort (Supplementary Fig. 1c). Western blot analysis revealed elevated WNT5B protein levels in striatal tissues from HD patients (Fig. 1c, d). Consistently, RNA-seq analysis of striatal tissue from R6/2 transgenic mice confirmed the upregulation of WNT5B (Fig. 1h, i). Immunofluorescence staining revealed that WNT5B expression was enriched in GFAP-positive astrocytes within both the caudate and putamen of HD patients (Fig. 1e–g), and colocalization analysis provided direct visual evidence of this overlap (Supplementary Fig. 1d). Similarly, increased WNT5B immunoreactivity was observed in S100β-positive astrocytes from N171-82Q mice (Fig. 1j, k). Finally, Western blot analysis revealed that, compared with wtHTT (25Q), transduction of mHTT (103Q) into human astrocytes significantly increased WNT5B protein levels (Fig. 1l–n). Collectively, these results indicate that mHTT induces WNT5B upregulation in astrocytes, which may contribute to the pathological mechanisms underlying HD.

### WNT5B overexpression alters the expression of ECM-related genes in HD mice

**Fig. 2:**
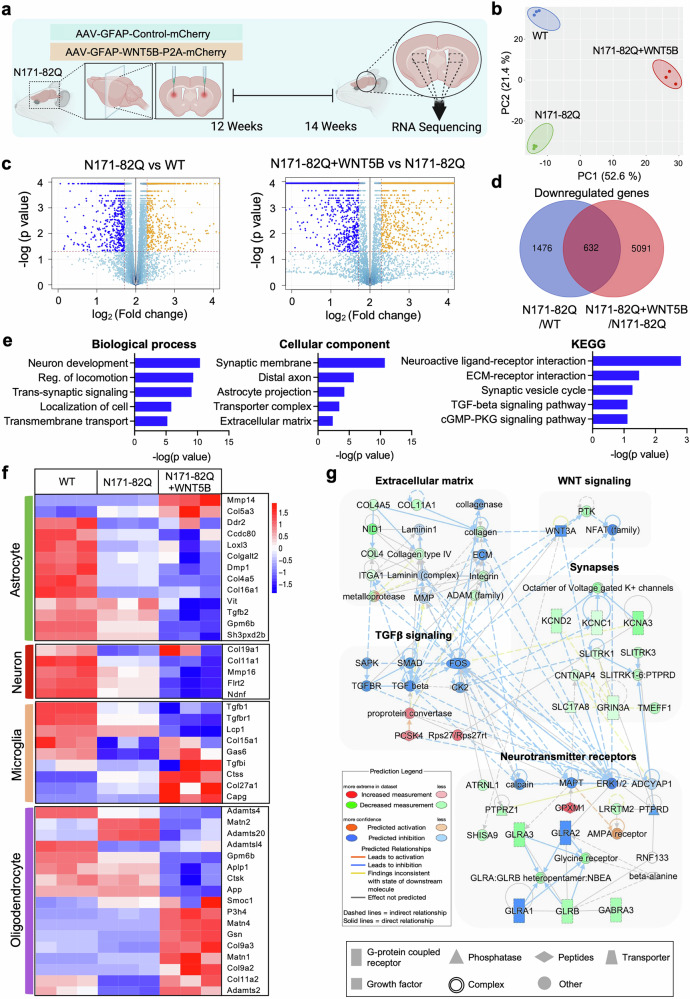
*WNT5B* overexpression alters ECM-related gene signatures in HD transgenic mice. (**a**) Experimental design showing the delivery of AAVs (pAAV-GFAP(pro)-Control-mCherry or pAAV-GFAP(pro)-WNT5B-P2A-mCherry) into the dorsal striatum of N171-82Q mice via bilateral stereotaxic injection at 12 weeks of age, followed by RNA-seq analysis of the striatal tissues from three groups of mice (WT, N171-82Q, and N171-82Q + WNT5B). **(b)** Principal component analysis (PCA) revealed distinct clustering of RNA-seq data among the three groups of mice (*N* = 3 mice per group). **(c)** Volcano plot showing differentially expressed genes in N171-82Q and N171-82Q + WNT5B mice. **(d)** Venn diagram illustrating the overlap of downregulated genes in N171-82Q mice whose expression was further decreased by WNT5B overexpression. **(e)** Gene ontology (biological process and cellular component) and KEGG analyses of the 632 genes downregulated in both N171-82Q and N171-82Q + WNT5B mice. **(f)** Heatmap showing alterations in the expression of cell type-specific ECM-related genes in WT, N171-82Q, and N171-82Q + WNT5B mice (*n* = 3 mice per group). **(g)** Ingenuity pathway analysis (IPA) revealed a molecular network linking WNT signaling with ECM-associated genes in N171-82Q and N171-82Q + WNT5B mice

To further investigate the mechanism underlying the gain-of-function effects of WNT5B on striatal astrocytes in HD, we delivered an AAV-GFAP-WNT5B-P2A-mCherry virus into the dorsal striatum of N171-82Q mice (N171-82Q + WNT5B) and performed RNA-seq on striatal tissue extracts **(**Fig. 2a**)**. The overexpression of *WNT5B* in astrocytes was verified by qPCR in human astrocytes and by immunostaining both in primary striatal astrocytes in vitro and in the striatum of injected mice **(**Supplementary Fig. 2**)**. Principal component analysis (PCA) demonstrated a distinct shift in transcriptome profiles induced by *WNT5B* overexpression in HD mice **(**Fig. 2b**)**. Volcano plots revealed the differentially expressed genes (DEGs) in N171-82Q and N171-82Q + WNT5B mice **(**Fig. 2c**)**. A total of 2,108 and 5,723 genes were downregulated in N171-82Q and N171-82Q + WNT5B mice, respectively **(**Fig. 2d**)**. We then performed gene ontology (GO) analysis via DAVID software on the 632 genes whose expression was downregulated in N171-82Q mice and whose expression was further suppressed by WNT5B overexpression. GO analysis revealed significant enrichment of extracellular matrix (ECM)-related processes among these genes, suggesting a deleterious effect of WNT5B overexpression on ECM integrity (Fig. 2e). The downregulation of multiple ECM-related genes in N171-82Q mice indicated ECM degradation in HDs, which was exacerbated by WNT5B overexpression. Among the astrocyte-specific ECM-related genes, *MMP14*, an ECM-degrading enzyme, was a key target that was markedly upregulated in N171-82Q + WNT5B mice (Fig. 2f and Supplementary Fig. 3b-d). Data from the Allen Institute Mouse Brain Atlas confirmed that both *WNT5B* and *MMP14* are expressed in striatal astrocytes^22^ (Supplementary Fig. 4). Next, we constructed a network model of the DEGs identified in both N171-82Q and N171-82Q + WNT5B mice via Ingenuity Pathway Analysis (IPA). The resulting network revealed dense interconnections among genes associated with critical biological pathways, including ECM organization, neurotransmitter receptor signaling, and TGFβ signaling (Fig. 2g).

### WNT5B overexpression induces MMP14 through the activation of noncanonical WNT signaling, which is suppressed by the phytoestrogen genistein

**Fig. 3:**
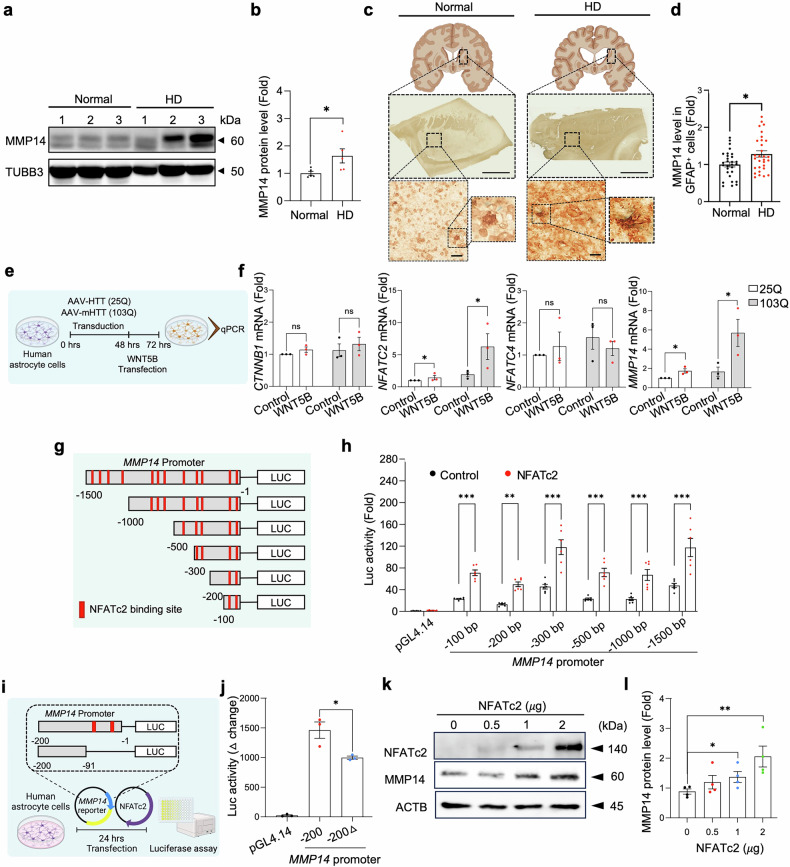
MMP14 levels are elevated in HD postmortem brains, and its expression is transcriptionally activated by NFATc2 through the noncanonical WNT signaling pathway in vitro. (**a**) Western blot analysis showing MMP14 protein levels in postmortem striatal tissues from normal subjects and HD patients. (**b**) Densitometry of the MMP14 protein levels (*n* = 5 samples per group). Student’s t test (*, *p* < 0.05). (**c**) Chromogenic staining for MMP14 (blue) and GFAP (brown) in postmortem striatal sections from normal subjects and HD patients. Scale bars: 20 μm. Whole-brain illustrations were created with BioRender.com. (**d**) Quantification of MMP14 immunoreactivity in GFAP-positive astrocytes in the putamen. (36 cells per group; 12 cells per case, *N* = 3 cases per group). Significantly different at *, *p* < 0.05. (**e**) Experimental design for the transfection of WNT5B into human astrocytes under normal (HTT-25Q) or HD (mHTT-103Q) conditions for 24 h, followed by mRNA expression analysis via qPCR. (**f**) *WNT5B* overexpression increased *NFATc2* and *MMP14* mRNA levels under both normal and HD conditions, whereas *CTNNB1* and *NFATc4* levels were unchanged. Significantly different at *, p < 0.05. (**g**) Schematic illustration of NFATc2-enriched binding sites (red) in the human *MMP14* promoter identified via the TRANSFAC 6.0-based Patch 1.0 algorithm. (**h**) Human *MMP14* promoter activity was analyzed in astrocytes transfected with serial deletion reporter constructs (-1500, -1000, -500, -300, -200, and -100 bp) for 48 h. Data represent three independent experiments (duplicates per experiment). Significantly different at **, *p* < 0.01; ***, *p* < 0.001. (**i**) Ex*p*erimental design for NFATc2 cotransfection with *MMP14* reporter constructs in human astrocytes for 24 h, followed by luciferase activity measurement. (**j**) The change in luciferase activity (Δ luciferase activity) for the *MMP14* promoter deletion construct (-200Δ) was significantly reduced compared with that of the full *MMP14* promoter (-200 bp). Significantly different at *, *p* < 0.05. (**k**) Western blot analysis showing the MMP14 *p*rotein levels in astrocytes treated with increasing doses of *NFATc2*. (**l**) Densitometry analysis of the MMP14 protein levels shown in panel (**k**). The data represent four independent experiments. One-way ANOVA (*, *p* < 0.05; **, *p* < 0.01)

Consistent with the transcriptomic data, Western blot analysis revealed a significant increase in total MMP14 protein in striatal extracts from HD patient postmortem brains compared with those from normal controls (Fig. 3a, b). In support of this observation, double chromogenic staining further confirmed increased MMP14 immunoreactivity in the striatal astrocytes of HD postmortem brain sections (Fig. 3c, d). Similarly, immunocytochemistry of primary mouse striatal astrocyte–neuron cocultures transduced with wtHTT (25Q) or mHTT (103Q) revealed a marked increase in MMP14 immunoreactivity in astrocytes following mHTT expression (Supplementary Fig. 5a-c). Western blot analysis further validated the increase in MMP14 protein levels in human astrocytes transduced with mHTT (Supplementary Fig. 5d).

To elucidate the mechanism by which WNT5B induces MMP14 expression, we performed a series of in vitro experiments. WNT5B signals through both the canonical (β-catenin-dependent) and noncanonical (β-catenin-independent) WNT pathways.^23^ To determine which pathway predominates under HD-related pathological conditions, we overexpressed WNT5B in AAV-mHTT (103Q)-transduced human astrocytes and analyzed the expression of *CTNNB1/β-catenin*, *MMP14*, and *NFAT* family genes via qPCR (Fig. 3e). WNT5B overexpression did not alter *CTNNB1* mRNA levels under either control (25Q) or HD (103Q) conditions (Fig. 3f). Immunocytochemical analysis also revealed no change in nuclear CTNNB1 (β-catenin) immunoreactivity, including the active (nonphosphorylated at Ser33/37/Thr41) form (Supplementary Fig. 6), indicating that WNT5B does not activate canonical WNT/β-catenin signaling in human astrocytes but rather preferentially engages a noncanonical pathway. Noncanonical WNT signaling activates NFAT transcription factors through Ca²⁺-dependent cascades.^24^ In this pathway, Ca²⁺-bound calmodulin activates calcineurin, which dephosphorylates NFAT proteins, promoting their nuclear translocation and transcriptional activation of their target genes.^25^ Among the five NFAT isoforms (NFATc1 ~ NFATc5), transcriptome analysis of the prefrontal cortex from HD patients revealed upregulation of *NFATc2* and *NFATc4* (Supplementary Fig. 7a). In human astrocytes, *WNT5B* overexpression significantly increased *NFATc2* expression under both normal (25Q) and HD (103Q) conditions (Fig. 3f), and immunostaining confirmed the nuclear translocation of active NFATc2 (Supplementary Fig. 8). Notably, treatment with KN-93, a CaMKII inhibitor that binds to calmodulin,^26^ significantly reduced nuclear NFATc2 accumulation in WNT5B-overexpressing astrocytes (Supplementary Fig. 8). By preventing the calmodulin‒calcineurin interaction, KN-93 inhibits calcineurin-mediated NFAT dephosphorylation and nuclear translocation. Together, these findings demonstrate that WNT5B preferentially activates noncanonical WNT/Ca²⁺ signaling in astrocytes under HD conditions, establishing a mechanistic link between WNT5B-mediated NFATc2 activation and MMP14 upregulation, which contributes to HD-related astrocytic pathology.

We predicted that the *MMP14* promoter contains approximately 12 NFATc2-DNA binding elements within the -1500 bp region of the 5’-UTR, as identified via the TRANSFAC 6.0-based Patch 1.0 algorithm^27^ (Supplementary Fig. 7b). To assess basal promoter activity and validate the functional relevance of these NFATc2-enriched binding sites, we transiently transfected human astrocytes with a series of nested deletion constructs encompassing progressive truncations of the *MMP14* promoter (-1500, -1000, -500, -300, -200, and -100 bp) alongside the pGL4.14 basic vector as a negative control (Fig. 3g). All reporter constructs exhibited NFATc2-dependent activation in human astrocytes, confirming that NFATc2-responsive elements are functionally present across the *MMP14* promoter region (Fig. 3h). To further localize the essential NFATc2-binding sites, we generated an additional deletion construct spanning the proximal region of the *MMP14* promoter (-90 to -1 bp) and constructed a luciferase reporter following transfection into human astrocytes (Fig. 3i). The *MMP14* promoter activity was markedly reduced in this truncated construct (-200Δ) compared with the full-length (-200) promoter role of the NFATc2 binding elements (Fig. 3j). Consistently, Western blot analysis revealed a dose-dependent increase in MMP14 protein levels upon NFATc2 overexpression in human astrocytes (Fig. 3k), with a significant increase detected at 1 µg of the *NFATc2* plasmid and further enhancement at higher doses (Fig. 3l). Similarly, immunostaining of primary mouse striatal astrocyte–neuron cocultures revealed increased astrocytic MMP14 immunoreactivity following NFATc2 overexpression (Supplementary Fig. 5e-f). To determine whether inhibition of NFATc2 activity attenuates MMP expression, we examined the effect of *Nfatc2* knockdown on MMP14 promoter activity via *Nfatc2*-targeting shRNA. As expected, sh*Nfatc2* significantly reduced MMP14 promoter-driven luciferase activity, a phenotype that was recapitulated by treatment with the phytoestrogen genistein (Supplementary Fig. 7f-g). Collectively, these results establish NFATc2 as a pivotal transcriptional regulator of *MMP14* expression in astrocytes, acting at both the mRNA and protein levels across multiple in vitro HD models.

### Estrogen receptor signaling antagonizes NFATc2-mediated transcription of MMP14 via physical interaction

**Fig. 4:**
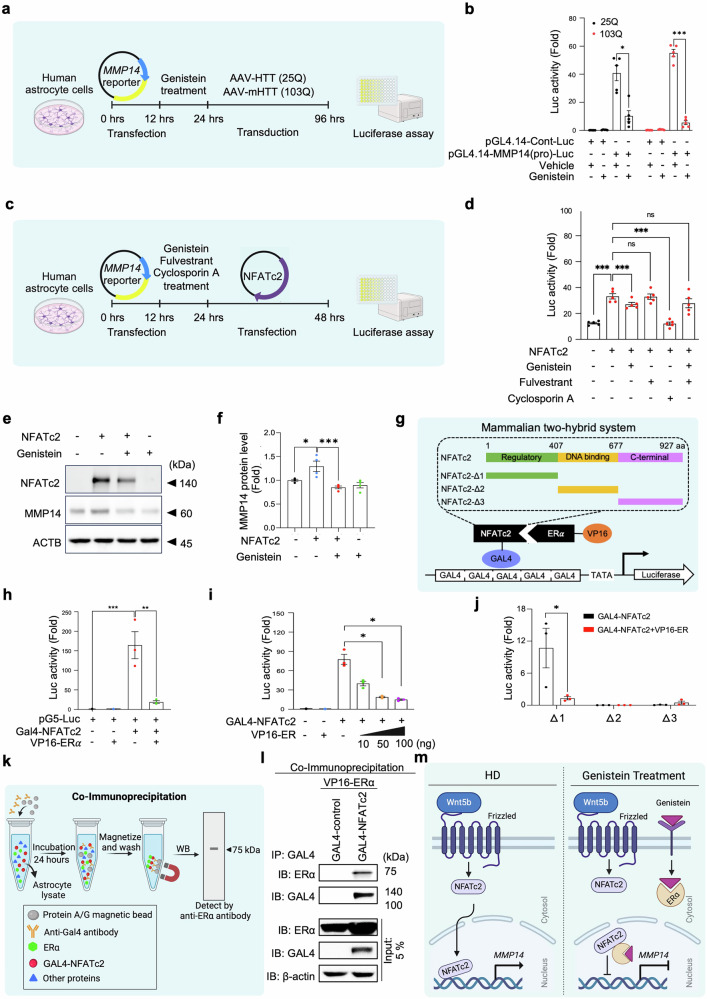
Phytoestrogen antagonizes NFATc2 transcriptional activity. (**a**) Experimental design for *MMP14* promoter transfection into human astrocytes under normal (HTT-25Q) or HD (mHTT-103Q) conditions, followed by genistein treatment and a luciferase reporter assay. **(b)** Genistein significantly reduced mHTT (103Q)-induced *MMP14* promoter activity (–200 bp construct). The data represent the means of five independent experiments. The error bars represent the means ± SEMs. One-way ANOVA (*, *p* < 0.05; ***, *p* < 0.001). **(c)** Experimental design for *MMP14* promoter transfection followed by treatment with genistein, fulvestrant (ER inhibitor), or cyclosporin A (NFAT inhibitor) under normal and HD conditions, with a subsequent luciferase assay. **(d)** Cyclosporin A decreased mHTT (103Q)-induced *MMP14* promoter activity (–200 bp), whereas fulvestrant blocked the genistein-mediated reduction in MMP14 promoter activity under HD conditions. The data represent the means of three independent experiments. One-way ANOVA (*, *p* < 0.05). **(e)** Western blot analysis of human astrocytes cotransfected with *NFATc2* and treated with genistein for 24 h. **(f)** Densitometry analysis of the MMP14 protein levels shown in panel (e). The data represent four independent experiments. One-way ANOVA (*, *p* < 0.05; **, *p* < 0.01). **(g)** Experimental design for mammalian two-hybrid interactions between NFATc2 and ER*α*. The full-length (amino acids 1 to 927), regulatory (amino acids 1 to 407), DNA-binding (amino acids 407 to 677), and C-terminal (amino acids 677 to 927) domains of NFATc2 were fused to the Gal4 DNA-binding domain (GAL4-NFATc2). ER*α* was fused to the VP16 transcriptional activation domain (VP16-ER*α*). **(h)** GAL4-NFATc2 alone increased luciferase activity, whereas cotransfection with VP16-ERα significantly reduced GAL4-NFATc2-induced luciferase activity. The data represent three independent experiments. **, *p* < 0.01; ***, *p* < 0.001. **(i)** VP16-ERα reduced luciferase activity in a dose-dependent manner. The data represent three independent experiments. *, *p* < 0.05. **(j)** NFATc2-△1 resulted in reduced luciferase activity upon cotransfection with VP16-ER. The data represent three independent experiments. *, *p* < 0.05. **(k)** Experimental workflow for immunoprecipitation (IP) analysis to detect NFATc2-ERα protein‒protein interactions. **(l)** ERα and NFATc2 interaction was verified by IP followed by Western blotting. **(m)** Proposed model illustrating the inhibition of the WNT5B‒NFATc2‒MMP14 axis by the genistein‒ERα interaction. WNT5B activates noncanonical WNT signaling, leading to NFATc2 and the induction of *MMP14* transcription. Genistein activates ERα, which physically interacts with NFATc2 and antagonizes its transcriptional activity. Schematic created with BioRender.com

The estrogen and WNT signaling pathways are known to converge at the level of their effector transcription factors.^28–30^ To examine the effect of genistein under pathological conditions in HD, we first performed an *MMP14* (-200 bp) promoter-luciferase assay in human astrocytes transduced with mHTT (103Q) (Fig. 4a). The increase in *MMP14* promoter activity observed in mHTT-expressing astrocytes was significantly attenuated by genistein treatment (Fig. 4b). This inhibitory effect was consistent across various promoter lengths containing NFATc2 binding sites and occurred in a dose-dependent manner (Supplementary Fig. 7c-e). To confirm the specificity of this action, we compared the effects of genistein with those of known inhibitors (Fig. 4c). The suppression of *MMP14* promoter activity by genistein was mimicked by the selective NFAT pathway inhibitor cyclosporin A and, notably, was completely abolished by cotreatment with the ERα antagonist fulvestrant (Fig. 4d). Similarly, MMP14 protein levels were significantly reduced in human astrocytes following genistein treatment (Fig. 4e, f). Immunocytochemical analysis further revealed that genistein effectively inhibited the WNT5B-induced nuclear translocation of active NFATc2 in human astrocytes (Supplementary Fig. 8).

To define the molecular mechanism underlying this antagonism, we employed a mammalian two-hybrid assay to assess how NFATc2 interacts with ERα **(**Fig. 4g**)**. Luciferase activity was robustly induced by GAL4-NFATc2 alone but was significantly suppressed upon cotransfection with VP16-ERα **(**Fig. 4h**)**, indicating that ERα represses NFATc2-driven transcriptional activity. Moreover, VP16-ERα inhibited luciferase activity in a dose-dependent manner **(**Fig. 4i**)**, further supporting the regulatory role of ERα in modulating NFATc2 activity. To identify the specific domain of NFATc2 responsible for ERα binding, we repeated the assay with NFATc2 deletion mutants. The results indicated that ERα interacts directly with the regulatory domain of NFATc2 **(**Fig. 4j**)**. This interaction was further confirmed by coimmunoprecipitation (IP), where NFATc2 pulled down with GAL4 was found to be complexed with ERα **(**Fig. 4k**)**. Western blot analysis of the IP products verified the ERα-NFATc2 interaction at the protein level, which was consistent with the mammalian two-hybrid findings **(**Fig. 4l**)**. Together, these results demonstrate that ERα physically associates with the regulatory domain of NFATc2 and antagonizes its transcriptional activity. Overall, WNT5B upregulation activated the β-catenin-independent signaling pathway to promote NFATc2-driven *MMP14* transcription, whereas genistein activated ERα, which directly interacts with NFATc2 to suppress *MMP14* transcription **(**Fig. 4m**)**.

### WNT5B gain of function disrupts ECM integrity and induces MSN damage in HD mice

**Fig. 5:**
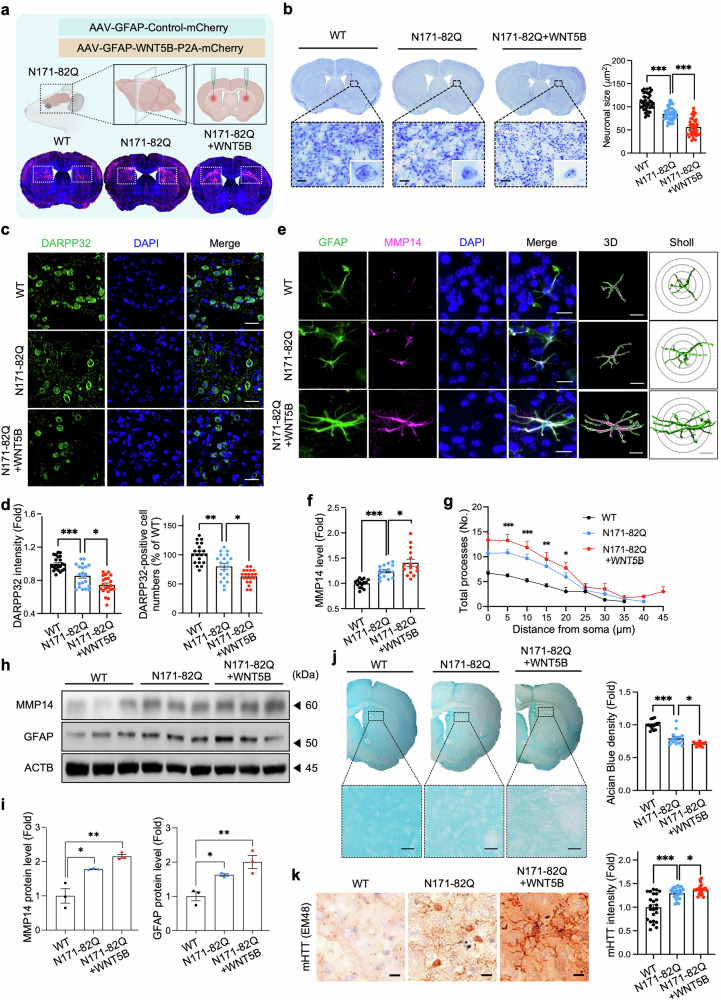
*WNT5B* overexpression enhances MMP14 expression in striatal astrocytes, leading to extracellular matrix (ECM) disruption and neuronal atrophy in HD mice. (**a**) Experimental design for AAV (pAAV-GFAP(pro)-Control-mCherry or pAAV-GFAP(pro)-WNT5B-P2A-mCherry) delivery into the dorsal striatum of N171-82Q mice via bilateral stereotaxic injection. (**b**) Brain sections stained with cresyl violet. Scale bars: 20 μm. Right: quantification of neuronal size in the dorsal striatum. A total of 40 cells per group were analyzed (8 cells per mouse; *N* = 5 mice per group: WT, N171-82Q, and N171-82Q + WNT5B). The error bars represent the means ± SEMs. One-way ANOVA (***, p < 0.001 ). (**c**) DARPP-32 immunoreactivity was reduced in the striatum of N171-82Q mice and further decreased in N171-82Q + WNT5B mice. Nuclei were counterstained with DAPI. Scale bars: 20 μm. (**d**) Quantitation of DARPP-32 immunoreactivity. A total of 20 cells per group were analyzed (4 cells per mouse; *N* = 5 mice per group: WT, N171-82Q, and N171-82Q + WNT5B). *, *p* < 0.05; **, *p* < 0.01; ***, *p* < 0.001. (**e**) Immunostaining for MMP14 and GFAP. Three-dimensional (3D) images were reconstructed for Sholl analysis of GFAP-positive astrocytes in the three groups of mice. Scale bars: 20 μm. (**f**) Quantification of MMP14 immunoreactivity in GFAP-positive cells in the dorsal striatum. A total of 15 cells per group were analyzed (3 cells per mouse; *N* = 5 mice per group: WT, N171-82Q, and N171-82Q + WNT5B). *, *p* < 0.05; ***, *p* < 0.001. (**g**) Quantification of the total number of processes associated with astrocytic processes via Sholl analysis. A total of 15 cells per group were analyzed (3 cells per mouse; *N* = 5 mice per group). One-way ANOVA (*, *p* < 0.05; **, *p* < 0.01; ***, *p* < 0.001). (**h**) Western blot analysis of the MMP14 and GFAP protein levels in striatal lysates from WT, N171-82Q, and N171-82Q + WNT5B mice. (**i**) Densitometry of MMP14 and GFAP protein levels from panel H. *N* = 3 samples per group. One-way ANOVA (*, *p* < 0.05; **, *p* < 0.01). (**j**) Alcian blue staining revealed the loss of glycosaminoglycans in the dorsal striatum of N171-82Q + WNT5B mice. Scale bar: 20 μm. Right: quantification of Alcian blue staining density in the dorsal striatum. Three ROIs (0.05 mm^2^ each) per mouse were analyzed (*n* = 5 mice per group). *, *p* < 0.05; ***, *p* < 0.001. (**k**) mHTT (mEM48) immunoreactivity was elevated in the striatum of N171-82Q + WNT5B mice. Scale bars: 10 μm. Right: quantification of mHTT immunoreactivity. A total of 25 cells per group were analyzed (5 cells per mouse; *N* = 5 mice per group). *, *p* < 0.05; ***, *p* < 0.001

To investigate how WNT5B gain-of-function affects HD pathogenesis in vivo, we delivered AAV-GFAP(pro)-WNT5B into the striatum of N171-82Q mice via bilateral stereotaxic injection **(**Fig. 5a**)**. The N171-82Q HD mouse model displays stable disease progression, a relatively long lifespan, and suitability for longitudinal behavioral and survival analyses, allowing extended assessment of neurodegenerative changes and therapeutic interventions.^31–33^ Immunofluorescence staining confirmed mHTT aggregation in the striatal astrocytes of N171-82Q mice **(**Supplementary Fig. 1e**)**. Nissl staining with cresyl violet (CV) revealed smaller nuclear sizes in medium spiny neurons (MSNs) **(**Fig. 5b**)**. Dopamine- and cAMP-regulated phosphoprotein 32 kDa (DARPP-32), which is expressed in more than 95% of MSNs, is known to be reduced in both HD patients and several HD mouse models.^34,35^ Consistent with these reports, DARPP-32 immunoreactivity and the number of DARPP-32-positive neurons were significantly lower in the striatum of N171-82Q + WNT5B mice than in that of N171-82Q mice **(**Fig. 5c, d**)**. Consistent with the in vitro results, MMP14 immunoreactivity was elevated in the striatal astrocytes of N171-82Q + WNT5B mice **(**Fig. 5e, f**)**. Furthermore, Western blot analysis of striatal lysates confirmed increased MMP14 protein levels in N171-82Q + WNT5B mice **(**Fig. 5h, i**)**. Alcian blue staining revealed a significant reduction in glycosaminoglycans (GAGs) in the striatum of N171-82Q, which was further exacerbated by WNT5B overexpression, indicating enhanced ECM degradation **(**Fig. 5j**)**. To further validate the pathological role of the WNT5B-MMP14 signaling pathway, we established an mHTT (103Q)-overexpressing mouse model by delivering AAV-mHTT (103Q) into the dorsal striatum of wild-type (WT) mice in parallel with control AAV-wtHTT (25Q) **(**Supplementary Fig. 9a**)**. Immunostaining with the EM48 antibody confirmed robust mHTT aggregation in the mHTT (103Q) mouse model **(**Supplementary Fig. 9b-c**)**. In this model, astrocytic WNT5B overexpression further increased MMP14 expression, exacerbated ECM degradation, and aggravated MSN atrophy **(**Supplementary Fig. 9d-k**)**.

In addition to MSN damage, the loss of parvalbumin-positive interneurons in the striatum is a recognized feature of HD pathology in both humans and rodents, contributing to dystonia and motor dysfunction.^36,37^ Parvalbumin-expressing interneurons in the striatum of N171-82Q + WNT5B mice presented significantly reduced Wisteria floribunda agglutinin (WFA) staining intensity, indicating the degradation of perineuronal nets (PNNs)–specialized ECM structures encapsulating these interneurons **(**Supplementary Fig. 10a**)**. Excessive MMP activity can degrade ECM components into proinflammatory fragments, thereby activating microglia and astrocytes.^38,39^ Compared with N171-82Q immunostaining, IBA1 immunostaining revealed increased microglial activation in N171-82Q + WNT5B mice (Supplementary Fig. 11a). GFAP immunostaining and Western blot analysis revealed pronounced astrocytic hypertrophy in N171-82Q + WNT5B mice compared with N171-82Q controls **(**Fig. 5e–i**)**. Moreover, immunostaining revealed increased levels of cleaved caspase-3, an active form of caspase-3, and reduced neuronal nuclear protein (NeuN) immunoreactivity in N171-82Q + WNT5B mice, confirming that WNT5B overexpression exacerbates neuronal apoptosis **(**Supplementary Fig. 12a-b**)**. Apoptotic signaling, in turn, promotes proteolytic cleavage of the mHTT protein into smaller aggregation-prone fragments, thereby accelerating both nuclear and cytoplasmic inclusion formation.^40^ Consistently, mHTT immunoreactivity was significantly elevated in the striatum of N171-82Q + WNT5B mice **(**Fig. 5k**)**. Together, these findings demonstrate that WNT5B overexpression enhances MMP14 expression in striatal astrocytes, leading to ECM degradation, mHTT aggregation, glial activation, and neuronal apoptosis in HD mice.

### WNT5B gain of function exacerbates motor deficits and shortens the lifespan of HD mice

**Fig. 6:**
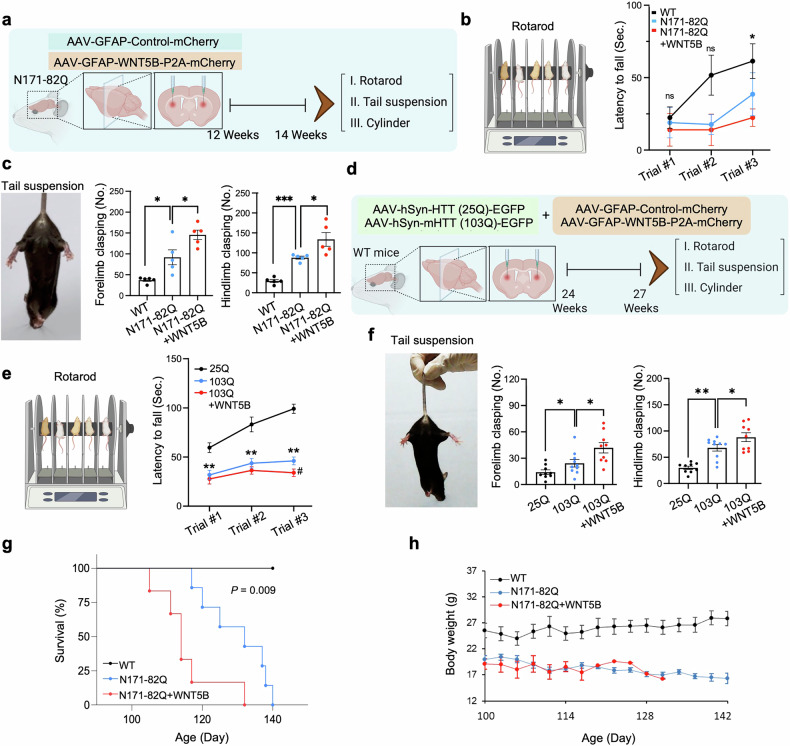
WNT5B gain of function exacerbates motor deficits and shortens the lifespan of HD mice. (**a**) Experimental design for the delivery of pAAV-GFAP(pro)-Control-mCherry or pAAV-GFAP(pro)-WNT5B-P2A-mCherry viruses into the dorsal striatum of N171-82Q mice via bilateral stereotaxic injection, followed by behavioral testing two weeks post-injection. **(b)** Latency to fall was reduced in N171-82Q + WNT5B mice during the rotarod test (*n* = 5 mice per group: WT, N171-82Q, and N171-82Q + WNT5B). The error bars represent the means ± SEMs. One-way ANOVA (Trial #1, *P* = 0.812; Trial #2, *P* = 0.129; Trial #3, *, *p* < 0.05). **(c)** The number of forelimbs and hindlimbs clasping during the tail suspension test at 2 weeks post-injection. *N* = 5 (WT, *N*171-82Q, N171-82Q + WNT5B). *, p < 0.05; ***, p < 0.001. **(d)** Experimental design for the delivery of AAV-HTT(25Q) (control) or AAV-HTT(103Q) (HD model) into the dorsal striatum of WT mice. For astrocyte-specific WNT5B overexpression, either pAAV-GFAP(pro)-Control-mCherry or pAAV-GFAP(pro)-WNT5B-P2A-mCherry was coinjected. Behavioral testing was conducted three weeks post-injection. **(e)** Rotarod test showing a reduced latency to fall in 103Q mice compared with 25Q controls, with the greatest impairment observed in 103Q + WNT5B mice (25Q, *N* = 9; 103Q, *N* = 10; 103Q + WNT5B, *N* = 9). One-way ANOVA (significantly different from 25Q mice at **, *p* < 0.01 and different from 103Q at ^#^, *p* < 0.05). **(f)** Tail suspension test at 2 weeks post-injection showing increased forelimb and hindlimb clasping in 103Q and 103Q + WNT5B mice (25Q, *N* = 9; 103Q, *N* = 10; 103Q + WNT5B, *N* = 9). *, *p* < 0.05; **, *p* < 0.01. **(g)** Kaplan‒Meier survival analysis showing that WNT5B overexpression significantly reduced the life span of N171-82Q mice (*n* = 7 mice per group: WT, N171-82Q, and N171-82Q + WNT5B). **(h)** Body weights of N171-82Q + WNT5B mice compared with those of N171-82Q and WT mice (*n* = 7 mice per group)

To evaluate the effects of WNT5B overexpression on motor coordination and activity in HD mice, we performed rotarod, cylinder, and tail suspension tests two weeks after delivering AAV-GFAP(pro)-WNT5B-P2A-mCherry into the striatum of N171-82Q mice via bilateral stereotaxic injection **(**Fig. 6a**)**. Compared with N171-82Q + WNT5B control mice, N171-82Q + WNT5B mice exhibited greater impairment of rotarod performance and motor coordination, indicating reduced motor coordination **(**Fig. 6b**)**. The number of forelimb and hindlimb clasps was significantly increased in N171-82Q + WNT5B mice in the TST, reflecting exacerbated motor imbalance **(**Fig. 6c**)**. In addition, the cylinder test results revealed that WNT5B overexpression significantly reduced spontaneous locomotor activity, such as rearing frequency, in N171-82Q + WNT5B mice. **(**Supplementary Fig. 15a**)**. To further confirm these findings with a larger sample size, we repeated the behavioral experiments in an AAV-mHTT (103Q)-induced HD mouse model **(**Fig. 6d**)**. In this model, AAV-HTT (25Q) served as a control, and AAV-mHTT (103Q) was injected into the dorsal striatum to induce HD-like pathology.^41^ Robust mHTT aggregation was confirmed in the dorsal striatum of 103Q mice via immunohistochemistry (IHC) with an EM48 antibody (anti-mHTT antibody) **(**Supplementary Fig. 9b-c**)**. Consistent with observations in N171-82Q mice, *WNT5B* overexpression further exacerbated motor deficits in 103Q HD mice, as demonstrated by decreased rotarod latency to fall **(**Fig. 6e**)** and increased clasping behavior in the tail suspension test **(**Fig. 6f**)**. K‒M survival analysis revealed a significantly shorter lifespan in N171-82Q + WNT5B mice (mean survival: 114 days) than in N171-82Q mice injected with the control virus (mean survival: 129 days) **(**Fig. 6g**)**. Body weight measurements revealed a progressive decrease beginning at approximately 125 days post-injection in N171-82Q + WNT5B mice **(**Fig. 6h**)**. Together, these findings demonstrate that gain of function of WNT5B in the striatum of both N171-82Q and AAV-mHTT (103Q)-induced HD mouse models exacerbates motor dysfunction and behavioral deficits and reduces survival, underscoring its pathogenic role in HD progression.

### Targeted knockdown of *Wnt5b*, *Nfatc2*, or *Mmp14* in astrocytes rescues ECM integrity and neuronal damage in HD mice

**Fig. 7:**
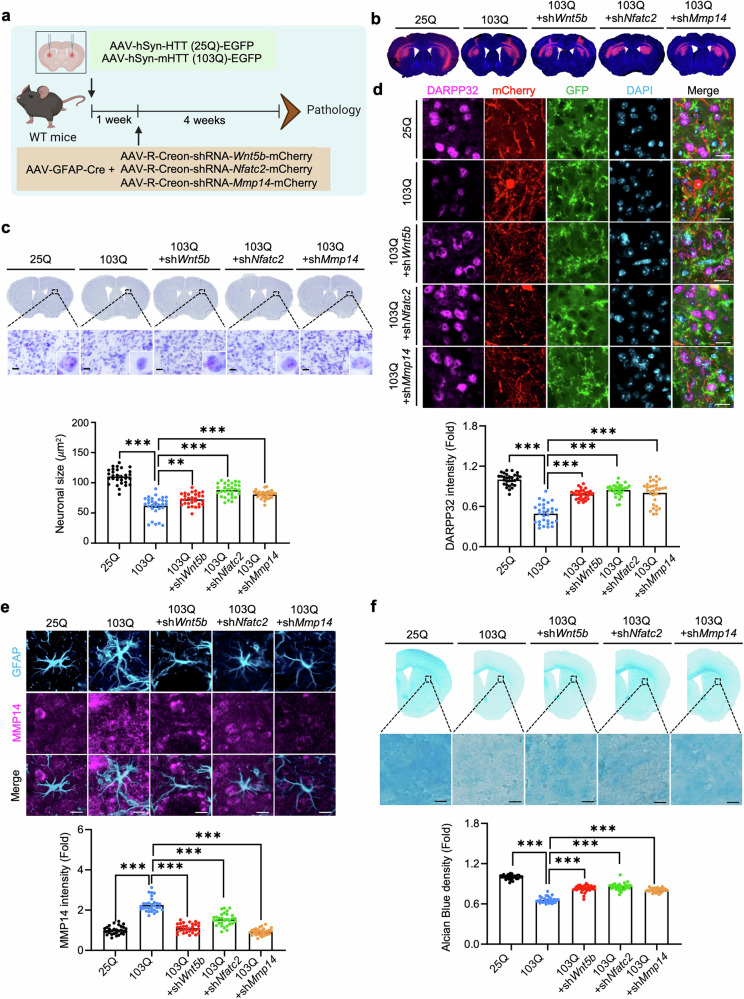
Astrocyte-specific knockdown of *Wnt5b, Nfatc2*, or *Mmp14* rescues ECM degradation and neuropathology in HD mice. (**a**) Experimental design for astrocyte-specific knockdown of *Wnt5b*, *Nfatc2*, or *Mmp14* with AAV-GFAP(pro)-Cre and Cre-dependent shRNA in the dorsal striatum of AAV-HTT (25Q) or AAV-mHTT (103Q) mice, followed by neuropathological assessment four weeks post-injection. **(b)** Representative brain sections confirming accurate delivery of AAVs into the dorsal striatum. **(c)** Cresyl violet (Nissl) staining of brain sections. Scale bars: 20 μm. Lower panel: quantification of neuronal size in the dorsal striatum. A total of 30 cells per group were analyzed (6 cells per mouse; *N* = 5 mice per group: 25Q-shCont, 103Q-shCont, 103Q-sh*Wnt5b*, 103Q-sh*Nftac2*, and 103Q-sh*Mmp14*). One-way ANOVA (**, *p* < 0.01; ***, *p* < 0.001). **(d)** Immunostaining and quantification of DARPP-32 immunoreactivity. Scale bars: 20 μm. A total of 30 cells per group were analyzed (6 cells per mouse; N = *5* mice per group (25Q-shCont, 103Q-shCont, 103Q-shWnt5b, 103Q-shNftac2, 103Q-shMmp14)). ***, *p* < 0.001. **(e)** Immunostaining and quantification of MMP14 intensity in GFAP-positive astrocytes. Scale bar: 20 μm. A total of 30 cells per group were analyzed (6 cells per mouse; *N* = 5 mice per group). ***, *p* < 0.001. **(f)** Alcian blue staining of the striatum showing the preservation of glycosaminoglycans following the knockdown of *Wnt5b*, *Nfatc2*, or *Mmp14*. Scale bars: 20 μm. Lower panel: quantification of Alcian blue density in the dorsal striatum. Six ROIs (0.05 mm^2^ each) per mouse were analyzed (*n* = 5 mice per group). ***, *p* < 0.001

To further validate whether the astrocytic WNT5B–NFATc2–MMP14 axis contributes to HD pathology in vivo, we employed an astrocyte-specific Cre-expressing AAV (AAV-GFAP-Cre) together with Cre-dependent shRNA vectors to selectively knock down *Wnt5b*, *Nfatc2*, or *Mmp14* in the striatum of an AAV-mHTT (103Q)-induced HD mouse model **(**Fig. 7a, b**)**. The knockdown efficiency of shRNAs targeting *Wnt5b*, *Nfatc2*, and *Mmp14* was validated by Western blotting and ICC in HEK^293T^ cells **(**Supplementary Table 4 and Supplementary Fig. 13**)**. Four weeks after the injection of shRNAs, neuronal morphology and ECM integrity were assessed in the striatum. Histological examination revealed that compared with 25Q + shCont mice, 103Q + shCont mice exhibited pronounced neuronal atrophy, as evidenced by a significant reduction in neuronal soma size **(**Fig. 7c**)**. In contrast, astrocytic knockdown of *Wnt5b*, *Nfatc2*, or *Mmp14* markedly rescued neuronal morphology, restoring neuronal size to near-control levels. Astrocytic knockdown of these genes also increased DARPP-32 immunoreactivity in the striatum of 103Q mice **(**Fig. 7d**)**. In parallel, MMP14 was strongly upregulated in GFAP-positive astrocytes from 103Q + shCont mice, whereas knockdown of *Wnt5b*, *Nfatc2*, or *Mmp14* significantly reduced the intensity of astrocytic MMP14 **(**Fig. 7e**)**. Alcian blue staining further demonstrated substantial loss of glycosaminoglycans in the striatum of 103Q + shCont mice, whereas silencing of *Wnt5b*, *Nfatc2*, or *Mmp14* preserved the glycosaminoglycan content, indicating protection of ECM integrity **(**Fig. 7f**)**. Collectively, these results indicate that astrocytic WNT5B–NFATc2–MMP14 signaling acts as a key pathological driver of ECM degradation and neuronal damage in HD. Targeted inhibition of this signaling pathway through astrocyte-specific gene silencing mitigates neurodegenerative changes and restores ECM homeostasis in vivo.

### Phytoestrogen prevents ECM degradation, reduces mHTT aggregation, and alleviates neuronal damage in HD mice

**Fig. 8:**
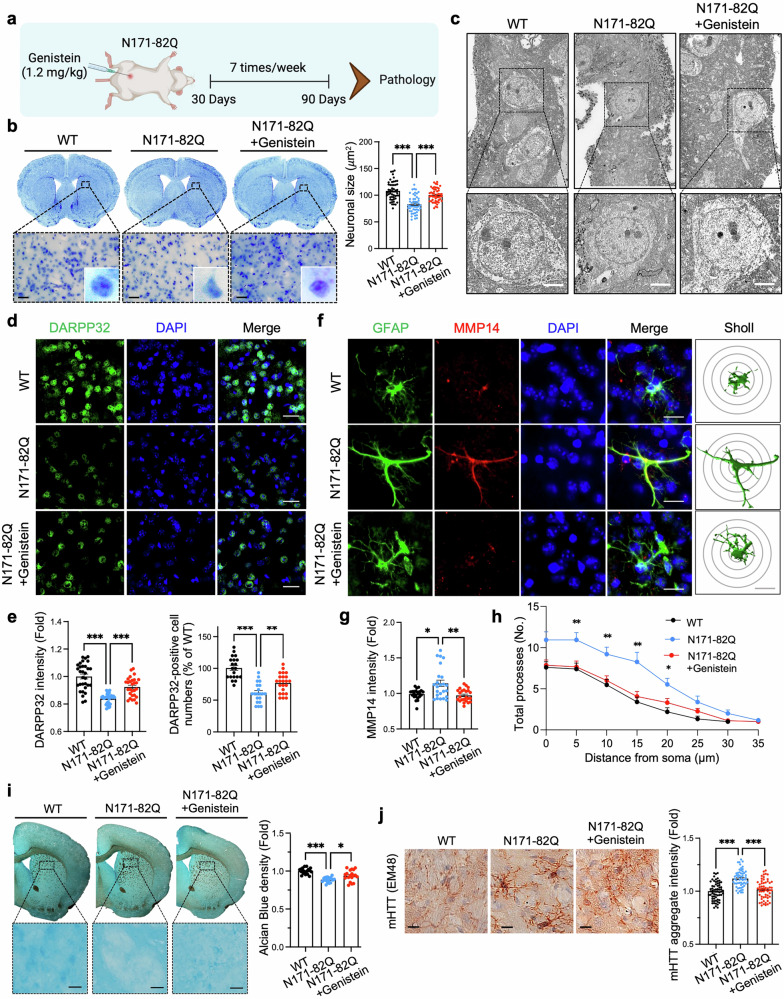
Genistein prevents ECM degradation, mitigates MSN damage, and reduces mHTT aggregation in the striatum of HD mice. (**a**) Experimental design for the treatment of N171-82Q mice with genistein via I.P. injection from postnatal days 30-90. (**b**) Cresyl violet (Nissl) staining of coronal brain sections. Right panel: quantitative analysis of neuronal soma size in the dorsal striatum. A total of 50 cells per group were analyzed (10 cells per mouse; *N* = 5 mice per group: WT, N171-82Q, and N171-82Q + Genistein). Scale bars (black): 20 μm. One-way ANOVA (***, p < 0.001). (**c**) Representative TEM images showing the restoration of MSN nuclear size by genistein treatment in N171-82Q mice. Scale bars: 2 µm. (**d**) Genistein treatment enhanced DARPP-32 immunoreactivity and increased the number of DARPP-32-positive neurons in the striatum of N171-82Q mice. Nuclei were counterstained with DAPI. Scale bars: 20 μm. (**e**) Quantification of the DARPP-32 signal and the number of DARPP-32-positive neurons. A total of 30 cells per group were analyzed (6 cells per mouse; *N* = 5 mice per group). **, *p* < 0.01; ***, *p* < 0.001. (**f**) Genistein reduced MMP14 and GFAP immunoreactivity in the striatum of N171-82Q mice. Scale bars: 20 μm. (**g**) Quantification of the MMP14 signal in striatal GFAP-positive cells. A total of 25 cells per group were analyzed (5 cells per mouse; *N* = 5 mice per group). *, *p* < 0.05; **, *p* < 0.01. (**h**) Quantification of astrocytic *p*rocesses via Sholl analysis. A total of 15 cells per group were analyzed (3 cells per mouse, *N* = 5 mice per group). The error bars represent the means ± SEMs. Student’s t test (*, *p* < 0.05; **, *p* < 0.01). (**i**) Alcian blue staining revealed preservation of glycosaminoglycans in the dorsal striatum of N171-82Q + genistein mice. Scale bar: 20 μm. Right panel: quantification of Alcian blue density in the dorsal striatum (four ROIs (0.05 mm^2^) per mouse, *N* = 5 mice per group). One-way ANOVA (*, *p* < 0.05; ***, *p* < 0.001). (**j**) Genistein reduced mHTT (mEM48) immunoreactivity in the striatum of N171-82Q mice. Scale bars: 10 μm. Right panel: Quantification of the mHTT signal in each group. A total of 60 cells were analyzed (12 cells per mouse; *N* = 5 mice per group). ***, p < 0.001

Genistein suppressed NFATc2 nuclear translocation and inhibited *MMP14* promoter activity in HD astrocytes **(**Fig. 4 and Supplementary Fig. 8**)**, thereby attenuating the WNT5B–NFATc2–MMP14 signaling pathway, which was previously shown to be pathogenic in vivo. To validate whether genistein affects *MMP14* transcription through antagonism of NFATc2 activity in vivo, genistein was administered intraperitoneally to N171-82Q mice (N171-82Q + genistein) daily from 30-90 days of age (1.2 mg/kg/day) **(**Fig. 8a**)**. Serial coronal brain sections revealed pronounced brain atrophy, bilateral ventricular enlargement, and flattening of the medial striatum in N171-82Q mice compared with WT controls. Notably, genistein treatment ameliorated these gross neuropathological alterations in N171-82Q mice **(**Fig. 8b**)**.

Nissl staining with cresyl violet (CV) confirmed that N171-82Q mice at 13 weeks of age presented marked striatal neuron atrophy, whereas genistein treatment significantly restored the nuclear size of MSNs, demonstrating a robust neuroprotective effect in genistein-treated N171-82Q mice **(**Fig. 8b**)**. Transmission electron microscopy (TEM) further revealed the shrinkage of MSN nuclei in N171-82Q mice and their apparent amelioration in genistein-treated mice (N171-82Q + genistein) **(**Fig. 8c**)**. Immunohistochemical analysis revealed that genistein treatment increased DARPP-32 immunoreactivity and the number of DARPP-32-positive neurons in the striatum of HD mice **(**Fig. 8d, e**)**. In parallel, MMP14 immunoreactivity in the striatal astrocytes of HD mice was reduced by genistein administration **(**Fig. 8f, g**)**. Consistently, GFAP immunostaining demonstrated attenuation of hypertrophic reactive astrocytes in N171-82Q + genistein mice **(**Fig. 8h**)**. Alcian blue staining further revealed the restoration of ECM integrity in N171-82Q + genistein mice **(**Fig. 8i**)**. Similarly, compared with AAV-mHTT (103Q) + vehicle control HD mice, AAV-mHTT (103Q)-induced HD mice treated with genistein presented reduced MMP14 expression, diminished ECM degradation, and preserved MSN morphology **(**Supplementary Fig. 14**)**. Notably, parvalbumin-positive interneurons presented a significant increase in the mean Wisteria floribunda agglutinin (WFA) staining intensity in genistein-treated mice, indicating recovery of encapsulating perineuronal net (PNN) structures **(**Supplementary Fig. 10b**)**. Moreover, IBA1 immunostaining revealed a reduction in the number of activated microglia in the striatum of N171-82Q + genistein mice **(**Supplementary Fig. 11b**)**. These findings suggest that genistein modulates glial activity, thereby reducing neuroinflammation and neuronal damage in HD mice. Consistent with these findings, cleaved caspase-3 immunoreactivity was significantly decreased in N171-82Q + genistein mice, confirming reduced neuronal apoptosis **(**Supplementary Fig. 12c**)**. Finally, mHTT immunoreactivity was markedly diminished in N171-82Q + genistein mice **(**Fig. 8j**)**. Together, these results demonstrate that genistein treatment mitigates neuronal apoptosis and neuroinflammation in HD mice by preventing ECM degradation through the suppression of astrocytic MMP14. Furthermore, genistein markedly reduces mHTT aggregation, contributing to overall neuroprotection in HD pathology.

### Phytoestrogen ameliorates motor deficits and extends lifespan in HD mice

**Fig. 9:**
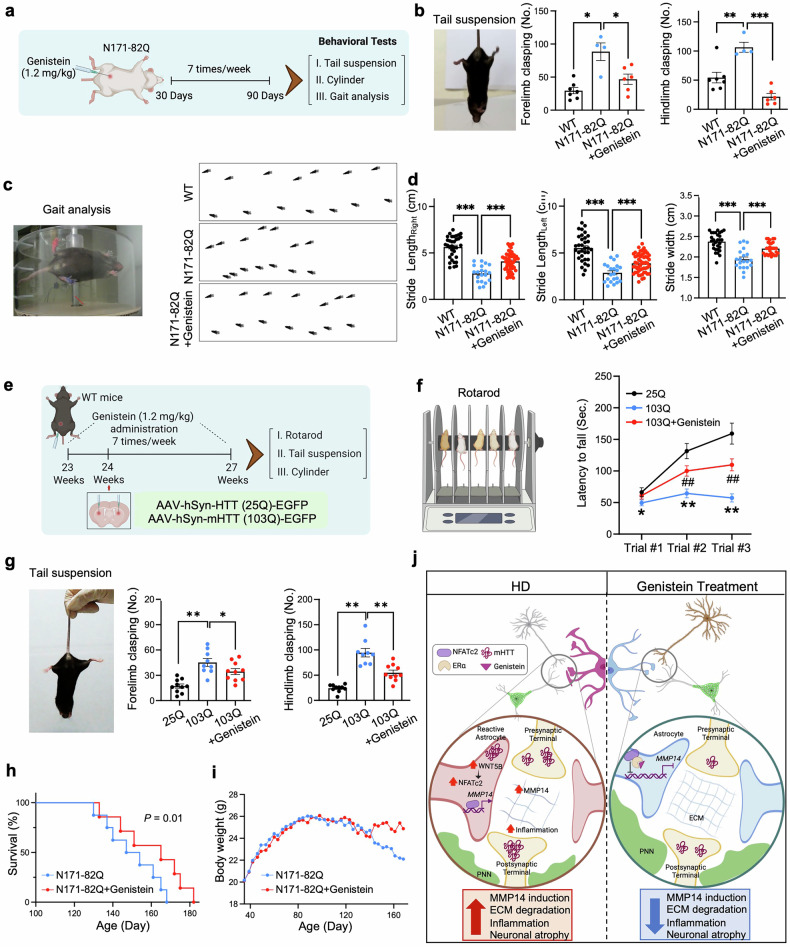
Genistein ameliorates motor deficits and extends lifespan in HD mice. (**a**) Experimental design for the behavioral assessment of N171-82Q mice treated with genistein or saline via I.P. injection from postnatal days 30 to 90. **(b)** Forelimb and hindlimb clasping behavior was quantified in the tail suspension test (WT, *N* = 7; N171-82Q, *N* = 4; N171-82Q + genistein, *N* = 6). One-way ANOVA (*, *p* < 0.05; **, *p* < 0.01; ***, *p* < 0.001). **(c)** Representative still images from the accelerated wheel test (left) and computer-assisted footprint analysis (right) in the wheel running paradigm across three experimental groups. **(d)** Genistein treatment improved stride length and stride width in N171-82Q (WT, *N* = 7; N171-82Q, *N* = 4; N171-82Q + genistein, *N* = 7). One-way ANOVA (***, *p* < 0.001). The data are presented as the means ± SEMs. **(e)** Experimental design for behavioral tests in AAV-mHTT (103Q)-induced HD mice following one month of i.p. administration of genistein or saline. **(f)** Rotarod performance showing that genistein significantly restored the latency to fall in 103Q mice relative to that in 103Q vehicle control mice (25Q, *N* = 10; 103Q, *N* = 9; 103Q + genistein, *N* = 10). One-way ANOVA (*, *p* < 0.05). **(g)** Tail suspension test showing that genistein alleviated forelimb and hindlimb clasping in 103Q mice compared with 103Q vehicle controls (25Q, *N* = 10; 103Q, *N* = 9; 103Q + genistein, *N* = 10). *, *p* < 0.05; **, *p* < 0.01; ***, *p* < 0.001. **(h)** Kaplan‒Meier survival analysis showing that genistein significantly prolonged the lifespan of N171-82Q mice (*n* = 10 mice per group: N171-82Q and N171-82Q + genistein). **(i)** Genistein treatment improved body weight maintenance in N171-82Q mice starting at 20 weeks of age. **(j)** Proposed model illustrating therapeutic modulation of the WNT5B-NFATc2-MMP14 signaling axis. Upregulation of WNT5B by mHTT activates noncanonical WNT signaling via NFATc2, inducing *MMP14* transcription in reactive astrocytes. Elevated MMP14 leads to ECM disruption, mHTT aggregation, and MSN damage. Genistein antagonizes NFATc2 activity and suppresses *MMP14* expression, thereby mitigating ECM degradation and neurodegeneration in HD. Schematic created with BioRender.com

We next investigated the effects of genistein on motor coordination and survival in HD model mice. N171-82Q mice received daily I.P. injections of genistein (1.2 mg/kg/d) from postnatal days 30-90, followed by tail suspension, cylinder, and gait analysis tests **(**Fig. 9a**)**. Genistein treatment markedly reduced forelimb and hindlimb clasping behavior in the tail suspension test **(**Fig. 9b**)** and improved spontaneous locomotor activity, as indicated by increased rearing frequency in the cylinder test **(**Supplementary Fig. 15b**)**.

To validate these findings in an independent HD mouse model with a larger sample size, we repeated the behavioral experiments with the AAV-mHTT (103Q)-induced HD model **(**Fig. 9e**)**. Consistent with the results from N171-82Q mice, genistein treatment consistently ameliorated motor deficits, as demonstrated by improved performance on the rotarod **(**Fig. 9f**)** and tail suspension **(**Fig. 9g**)** tests.

Computer-assisted footprint analysis was employed to evaluate gait during the wheel running task **(**Fig. 9c**)**. Quantitative analysis of footprint patterns revealed improved motor coordination, as evidenced by normalized stride length and stride width in N171-82Q + genistein mice **(**Fig. 9d**)**. Importantly, these behavioral improvements paralleled neuropathology recovery and translated into prolonged survival in N171-82Q mice, with a 19% increase in lifespan (median survival: 105 days in N171-82Q versus 125 days in N171-82Q + genistein; χ^2^ = 9.23; *p <*= 0.01) **(**Fig. 9h**)**. Furthermore, genistein significantly mitigated the decrease in body weight observed in N171-82Q mice (F_(4.60)_ = 7.910; *P* = 0.009) **(**Fig. 9i**)**. Collectively, the results of the behavioral analyses demonstrated that genistein treatment restored impaired motor coordination and extended survival in HD model mice.

Mechanistically, under HD conditions, mHTT induces WNT5B expression in astrocytes, activating the noncanonical WNT signaling pathway and promoting NFATc2-mediated transcription. NFATc2 activation upregulates *MMP14* expression in reactive astrocytes. Excess MMP14 accelerates ECM degradation, neuronal atrophy, and mHTT aggregation. Genistein enhances ER*α* activity, which physically interacts with NFATc2 and antagonizes its transcriptional activity at the *MMP14* promoter. Consequently, the suppression of *MMP14* expression by genistein prevents ECM degradation and reduces neuronal atrophy **(**Fig. 9j**)**.

## Discussion

In this study, we identified a novel astrocytic WNT5B‒NFATc2‒MMP14 signaling axis as a critical pathological cascade driving ECM degradation and neuronal atrophy in HD. Our findings revealed that WNT5B, a noncanonical WNT ligand, is aberrantly upregulated in reactive astrocytes of both HD patients and HD model mice, where it activates NFATc2 to induce MMP14 expression. This mechanism establishes WNT5B as a direct upstream regulator of NFATc2-mediated MMP14 transcription, linking dysregulation of WNT signaling to astrocyte-driven ECM remodeling and neurodegeneration.

While previous studies have implicated general WNT signaling abnormalities in neurodegenerative disease,^42,43^ our work specifically delineates the noncanonical WNT5B‒NFATc2 pathway as a bona fide mechanism underlying HD pathology and identifies it as a potential therapeutic target for HD and related neurodegenerative diseases.^43^ Both the knockdown of WNT ligands and the downstream transcription factor pangolin/TCF ameliorate survival in the *Drosophila* HD model.^44^ In this study, we discovered that *WNT5B* is upregulated in the striatal astrocytes of HD patients and HD model mice. We also verified that mHTT increased WNT5B and NFATc2 protein levels in human astrocytes. The upregulation of *WNT5B* consequently activated the NFATc2 transcription factor, among other NFAT family members, through a noncanonical WNT signaling pathway. The activation of WNT/Ca^2+^ signaling leads to the dephosphorylation and subsequent nuclear translocation of NFAT, which triggers the transcriptional activation of NFAT target genes.^25^ We analyzed the whole transcriptome and revealed that the genes whose expression was most significantly downregulated by WNT5B overexpression in HD mice were associated with TGFβ signaling and synapse pathways. TGFβ1 is a well-known anti-inflammatory cytokine in brain glia.^45^ The downregulation of TGFβ signaling by WNT5B overexpression in our data, combined with the known anti-inflammatory effects of TGFβ1 in the brain, suggests that impaired TGFβ signaling may trigger a shift toward a more proinflammatory state in HD.^46,47^ On the other hand, the ECM coordinates the formation and maintenance of neural circuitry under normal conditions, and its dysfunction and degradation increase the risk for various neurological disorders.^48^ The ECM degradation enzyme *MMP14* was the most significantly upregulated gene among the ECM components in HD mice. Moreover, the *MMP14* promoter region contains several NFATc2 binding sites that activate *MMP14* transcription. We confirmed that WNT5B overexpression induces *MMP14* at both the mRNA and protein levels in an NFATc2 transcription factor-dependent manner through the noncanonical WNT signaling pathway. MMP14 is a member of the matrix metalloprotease enzyme family that facilitates local ECM degradation.^49,50^ *MMP10* and *MMP14* are upregulated in Hdh^111Q/111Q^ mouse striatal cells compared with Hdh^7Q/7Q^ cells, and MMP10 directly cleaves the huntingtin protein into toxic N-terminal fragments.^51^ Moreover, siRNA-mediated knockdown of *MMP10* and *MMP14* in Hdh^111Q/111Q^ cells effectively blocked caspase-3/7 activity. This resulted in a reduction in apoptosis.^51^

Our use of the N171-82Q model, although atypical for astrocyte-centered studies, was a strategic choice. This model results in slower and more stable disease progression, enabling extended longitudinal analyses of glial and neuronal interactions. We validated its suitability by confirming the presence of mHTT aggregates within S100β-positive astrocytes, which is consistent with prior reports of noncell autonomous, prion-like propagation of mHTT from neurons to glial cells.^32,33^ These findings substantiate the N171-82Q model as a valid experimental system for dissecting astrocyte-mediated mechanisms of pathology in HD. mHTT modulates epigenetic modifications and transcriptional pathways in MSNs and astrocytes in HD.^52^ In this study, we confirmed that the *MMP14* gene was induced at the protein level by transducing mHTT in astrocytes. MMP14 is secreted into the extracellular space through microvesicular exosomes.^53^ Excess MMP14 in the ECM leads to local ECM and PNN degradation into small fragments, which trigger proinflammatory signals and activate astrocytes and microglia.^38^ The cell-autonomous ECM and actin cytoskeletal signaling pathways are abnormally altered in HD patient-derived astrocytes and subsequently lead to changes in cell morphology and adhesion.^54^ ECM degradation is a well-known factor that activates microglia, which are characterized by an ameboid morphology, large and round cell bodies, and thick protrusions that secrete proinflammatory signals.^38^ A previous study suggested that the depletion of microglia may be a plausible therapeutic approach for preventing ECM degradation in an HD mouse model.^55^ While the microglial activation response is initially protective in acute and mild stress models, chronic activation of microglia leads to neuroinflammation and neuronal atrophy, which expedites neurodegeneration.^56^ Our data indicate that MMP14 induction by WNT5B and ECM degradation is associated with an increase in the number of IBA1-positive active microglia and inflammatory responses in the striatum of HD mice. On the other hand, from the viewpoint of neuronal damage, we confirmed that WNT5B overexpression significantly elevated the level of active/cleaved caspase-3, a key marker of apoptotic neuronal cell death, in the striatal MSNs of HD mice. Moreover, WNT5B expression increased mHTT aggregation in the striatal MSNs of HD mice. The chain reaction triggered by the WNT5B-MMP14 pathway likely exacerbates a vicious cycle of neuronal damage and promotes neurodegeneration in HD mice. Overall, we propose that the WNT5B-dependent molecular and cellular pathways contribute to impaired motor behaviors, such as abnormal gait and hypokinesis, and shorten the lifespan of HD mice.

Increasing evidence has shown that phytoestrogens prevent neuronal damage and delay the progression of neurodegenerative disorders.^20^ Genistein is the most active estrogenic compound present in soy.^57^ Pretreatment with genistein significantly reduces the formation of Aβ and Aβ-induced neurotoxicity in primary rat hippocampal neurons.^58^ As a mode of action, genistein drives epigenetic alterations by activating an estrogen receptor (ER) in both in vitro and in vivo studies.^59^ It modulates gene expression throughout the body of mice in a dose- and time-dependent manner at all ages by activating ERs.^60^ Genistein is known to influence multiple cell types in the brain, including neurons, microglia, and astrocytes, through its pleiotropic actions, such as the modulation of estrogen receptors, antioxidant pathways, and anti-inflammatory signaling.^61,62^ In addition, accumulating evidence suggests that genistein exerts cell type-specific effects and that astrocytes are particularly responsive to genistein through transcriptional pathways.^63,64^ From the perspective of transcriptional regulation, it has been shown that ER represses NFATc4 transcriptional activity.^29^ In this study, WNT5B robustly induced NFATc2 in astrocytes, whereas genistein antagonized its transcriptional activity. Genistein activates ERα, which directly interacts with the NFATc2 regulatory domain, thereby suppressing NFATc2-dependent transcription. These findings identify a therapeutic mechanism in which genistein reduces *MMP14* expression by repressing NFATc2 activity via the noncanonical WNT signaling pathway.

In summary, our current preclinical study revealed that genistein administration at the presymptomatic stage repressed *MMP14* expression in astrocytes and prevented ECM degradation in the striatum of HD mice. Genistein administration also reduced neuroinflammation, mHTT aggregation, and damage to MSNs in the striatum of HD mice. Consequently, genistein administration improved the motor behaviors of HD mice, prevented body weight loss, and prolonged the lifespan of HD mice. Despite extensive research efforts to treat neurodegenerative disorders, no drugs targeting the WNT signaling pathway have been approved for clinical use.^13^ Taken together, our findings suggest that genistein is a small compound that targets the noncell-autonomous WNT5B-NFATc2-MMP14 signaling pathway and delays the progression of HD pathogenesis.

## Methods

### Human brain samples

Neuropathological examination of normal and HD postmortem brain samples followed established protocols from the Boston University Alzheimer’s Disease Research Center (BU ADRC). Striatal histopathology was confirmed and graded using criteria from a previous study.^65^ The Institutional Review Board (IRB) of the BU School of Medicine exempted this study (IRB protocol number H-28974) because it utilized postmortem tissues not classified as human subjects.^21^ The research complied with institutional regulatory guidelines and the principles of the Declaration of Helsinki. All the information about the specimen was protected by the BU ADRC system in accordance with NIH policy. Details of the brain tissues are provided in Supplementary Table 1.

### Animals

Male and female N171-82Q HD transgenic mice were obtained from The Jackson Laboratory (Bar Harbor, ME, USA) and bred as colonies in the KIST SPF Animal Facility. Detailed mouse information is provided in Supplementary Table 3. At 12 weeks of age, the mice received injections of AAV-GFAP(pro)-WNT5B-P2A-mCherry virus via a stereotaxic microinjection device (Stoelting Co.). AAVs were administered into the striatum (coordinates: AP: 0.86 mm, ML: +/- 2 mm, DV: 2.9 mm) of both wild-type and HD transgenic mice as previously described, whereas the control groups received AAV-scrambled-shRNA injections. Genistein (Sigma, USA) was administered (1.2 mg/kg, i.p. injection) to wild-type and N171-82Q mice from 30-90 days of age, seven times per week. The control groups were injected with saline. Behavioral, neuropathological, and biochemical analyses were conducted two weeks post-injection. Deaths occurring overnight were recorded the following morning. Kaplan‒Meier survival plots were generated on the basis of the last survival date or the date of euthanasia if the mice could not initiate movement or right themselves after 30 seconds of gentle prodding. The mice were kept on a 12:12-hour light‒dark cycle (lights on at 8:00 AM) with ad libitum access to food and water. All procedures involving animals were conducted in accordance with the guidelines approved by the Institutional Animal Care and Use Committee (IACUC) and adhered to the animal welfare regulations of KIST (Seoul 02792, South Korea).

### Cell cultures

#### Primary striatal cultures

Striatal tissue was harvested from postnatal day 0–3 (P0–P3) C57BL/6 mice, following procedures previously reported.^66^ Dissected striata were carefully cleared of meninges, minced, and subsequently dissociated into single cells by gentle trituration. The resulting suspension was plated onto poly-D-lysine–coated surfaces (0.1 mg/mL; Sigma, USA). Cells were maintained in Neurobasal medium (Thermo Fisher Scientific, USA) enriched with 25 mM L-glucose, 10% heat-inactivated horse serum, 10% heat-inactivated FBS, B-27 supplement (20 mM), FUDR (10 mM), and penicillin-streptomycin (1000 U/mL). Cultures were incubated at 37 °C with 5% CO₂ under humidified conditions. On day 3 in vitro (DIV3), cultures were washed thoroughly by pipetting and replenished with fresh medium to remove cellular debris and non-attached cells.

#### Human astrocyte line

The immortalized human astrocyte cell line (fetal-SV40) was obtained from Applied Biological Materials Inc. (Cat. No. T0280, Canada). Cells were cultured in DMEM supplemented with 25 mM glucose, 10% heat-inactivated FBS, 2 mM glutamine, and penicillin-streptomycin (1000 U/mL). For experiments, astrocytes at passages 10–20 were used.

### Chemicals

For in vitro pharmacological experiments, the following small-molecule inhibitors were employed: KN-93 (CaMKII inhibitor; Sigma, K1385, USA) at a final concentration of 1 μM; cyclosporin A (NFAT pathway inhibitor; Sigma, SML1018, USA) used at 0.25 μM; and fulvestrant (estrogen receptor α/β antagonist; Sigma, I4409, USA) applied at a working concentration of 100 nM.

### Mammalian two-hybrid luciferase assay

The CheckMate™ Mammalian Two-Hybrid System (E2440, Promega) was used to verify the interaction between NFATc2 and ER*α*. The pG5luc vector was used to assess luciferase expression mediated by the interaction between GAL4-NFATc2 and VP16-ER*α*. Human astrocytes were transiently transfected with pG5luc, pBIND-NFATc2, or pACT-ER*α*, followed by a 48-hour incubation to allow stabilization. To assess luciferase activity, the cells were washed with PBS, lysed with 5X lysis reagent, and detected via the luciferase assay buffer provided in the Luciferase Assay System (E1500, Promega).

### Quantitative real-time PCR (qPCR)

Total RNA was extracted from cultured cells and mouse brain tissue using a Qiagen RNA isolation kit (Qiagen, USA). Complementary DNA (cDNA) was synthesized from 1 μg of total RNA using the iScript cDNA Synthesis Kit (Bio-Rad, USA), following the manufacturer’s protocol. Quantitative PCR was carried out using iQ SYBR Green Supermix (Bio-Rad, USA), and gene expression levels were normalized to GAPDH mRNA. Thermal cycling was performed under the following conditions: an initial denaturation at 95 °C for 3 min, followed by 40 amplification cycles consisting of 95 °C for 15 s, 60 °C for 15 s, and 70 °C for 20 s, with a final extension at 72 °C for 30 s. Melting curve analysis was generated using 33 cycles of 6 s each, beginning at 60 °C and increasing to 92 °C, with increments of 1 °C after the second cycle. The primer sequences used were: NFATc2: F 5’-TGCGGAAGCCACCAGGAGTT-3’, R 5’-TTGGCGGCTCTTTGGCTCGT-3’; NFATc4: F 5’-GCACCGTATCACAGGCAAGATG-3’, R 5’-TCAGGATTCCCGCGCAGTCAAT-3’; MMP14: F 5’-AGGCCATTCGCAAGGCGTTC-3’, R 5’-ACCATCGAAGGGCGTGCTGT-3’; CTNNB1 (β-catenin): F 5’-TGGCCCAGAATGCAGTTCGC-3’, R 5’-TGGCACCCTGCTCACGCAAA-3’.

### Immunofluorescence labeling of cultured cells

Cultured cells were washed twice with PBS prior to fixation in 4% paraformaldehyde (PFA) for 10 min at room temperature. After fixation, cells were rinsed again in PBS and permeabilized with 0.1% Triton X-100 in PBS for 15 min. To reduce nonspecific antibody binding, cells were incubated for 2 h at room temperature in 3% bovine serum albumin (Sigma, USA) or normal goat/donkey serum (Abcam, USA) prepared in PBS containing 0.1% Triton X-100. Primary antibody incubation was performed overnight (24 h) at 4 °C, using the following antibodies at the indicated dilutions: WNT5B (1:100, Santa Cruz, USA), MMP14 (1:200, Abcam, USA), NFATC2 (1:100, Invitrogen, USA), S100β (1:200, Synaptic Systems, Germany), β-catenin (1:200, Santa Cruz, USA), and non-phospho (active) β-catenin (Ser33/37/Thr41; 1:1000, Cell Signaling, USA). After incubation with fluorophore-conjugated secondary antibodies, samples were rinsed three times in PBS and visualized using a confocal microscope.

### Mouse brain tissue processing and confocal microscopy

Mice were anesthetized deeply with 2% avertin (20 mg/mL; 20 μL per g body weight, intraperitoneally) and transcardially perfused with 0.9% saline, followed by ice-cold 4% paraformaldehyde (PFA). Brains were removed, postfixed in 4% PFA at 4 °C overnight, and cryoprotected sequentially in 15% and 30% sucrose solutions for 48 h. Coronal striatal sections (30 μm thickness) were cut using a cryostat and preserved in storage buffer at 4 °C until use. Free-floating sections were rinsed in PBS and incubated for 1 h in a blocking/permeabilization buffer consisting of 0.3% Triton X-100 and 2% normal donkey serum in 0.1 M PBS. Tissue was then exposed to primary antibodies diluted in the same blocking solution and allowed to incubate overnight at 4 °C on a shaker. After washing three times with PBS, sections were treated with fluorophore-conjugated secondary antibodies for 1 h at room temperature, followed by an additional PBS wash. DAPI (1:5,000, Abcam, USA) was applied during the second wash for nuclear labeling. Slides were mounted using fluorescent mounting medium (Dako, USA) and air-dried at room temperature. Images were acquired using a Nikon A1 confocal microscope (Nikon, Japan) with Z-stacks collected at 26 μm depth using 2 μm steps. Image processing and Sholl analysis were performed using NIS-Elements (v4.5, Nikon) and ImageJ (v1.52 s, NIH). Primary antibodies used: GFAP (1:200, MAB3402, Millipore), mCherry (1:500, Abcam), GFP (1:200, Santa Cruz), WNT5B (1:100, Santa Cruz), NFATC2 (1:100, Invitrogen), MMP14 (1:200, Abcam), DARPP32 (1:200, Abcam), S100β (1:500, Synaptic Systems), cleaved caspase-3 (1:200, Cell Signaling), Iba1 (1:400, Wako), mHTT (1:100, MAB5374, Millipore), and Wisteria floribunda agglutinin (WFA) lectin (1:200, Vector Labs). Secondary antibodies were diluted 1:200 in blocking solution and applied for 2 h at room temperature.

### Wisteria floribunda agglutinin (WFA) staining

For WFA staining, free-floating sections were rinsed with TBS before beginning the histological procedures. To block nonspecific binding sites, the sections were incubated for 1 hour in a solution of 5% normal donkey serum in TBS containing 0.3% Triton X-100. The sections were then incubated overnight with biotinylated WFA (10 µg/ml, 1:200, B-1355-2, Vector Labs). Following incubation with a cocktail containing Alexa Fluor™ 594 streptavidin (10 µg/ml, 1:200, S11227, Invitrogen) in TBS containing 2% bovine serum albumin (TBS-BSA) for 2 h at room temperature, the sections were thoroughly rinsed with TBS, briefly rinsed in distilled water, mounted on fluorescence-free slides, air-dried, and cover-slipped.

### Alcian blue staining

The sections were rinsed twice with distilled water. An Alcian blue solution was prepared following the manufacturer’s protocol for staining. First, we incubated the sections with 3% (v/v) glacial acetic acid for 3 minutes at room temperature. Then, we stained the sections with 1% Alcian blue solution (TMS-010-C, Merck) for 30 minutes. Following incubation, the staining solution was removed, and the sections were washed to eliminate excessive stain. Finally, the slides were mounted and examined under a microscope. Three images per mouse were randomly selected for optical density quantification, and measurements were performed via ImageJ.

### Dual chromogenic staining in human postmortem brain sections

#### First staining step

Coronal sections (10 µm thick) were prepared from paraffin-embedded postmortem striatal tissues obtained from five healthy controls and five patients with HD. Endogenous alkaline phosphatase activity was quenched using BLOXALL® Blocking Solution (SP-600, Vector Laboratories, USA). Sections were then incubated in 5% bovine serum albumin (A5611, Sigma-Aldrich, USA) for 1 h to block nonspecific binding, followed by exposure to anti-GFAP antibody (1:200, Millipore, USA) for 24 h. After washing three times with PBS, signal amplification was performed using the Vector ABC kit **(P**K-4000, Vector Laboratories, USA), and GFAP labeling was visualized with DAB substrate (D7304, Thermo Fisher Scientific, USA). Second staining step: After GFAP development, the same slides were incubated with anti-MMP14 antibody (1:200, Abcam, USA) for 24 h. Detection was carried out using ImmPRESS-AP anti-rabbit IgG polymer reagent (MP-5401, Vector Laboratories, USA) for 2 h at room temperature. Vector Blue alkaline phosphatase substrate (SK-5300, Vector Laboratories, USA) was applied to reveal MMP14 staining. The stained sections were sequentially dehydrated through graded ethanol solutions (70%, 80%, 90%, 95%, and 100%), cleared in Histo-clear (HS-200, National Diagnostics, USA), and coverslipped. Staining signals—GFAP in brown and MMP14 in blue—were examined using a BX63 light microscope (Olympus, Japan) equipped with a DP74 1920×1200-pixel digital camera (Olympus, Japan).

### Transmission electron microscopy (TEM)

TEM was performed following the method described by Lee et al.^67^. The brain samples were fixed for 1 hour in a solution containing 2% glutaraldehyde, 0.2% freshly prepared tannic acid, and 0.1 M sodium cacodylate (pH 7.4). After washing with cacodylate, the samples were postfixed in 0.5% OsO_4_ and embedded in Durcupan (Fluka, Switzerland). Thin sections were then prepared, contrasted with uranyl acetate and lead citrate, and examined via an 80 kV transmission electron microscope.

### AAV production and stereotaxic injection

Multiple AAV vectors were used in this study. For HTT expression, two distinct sets of vectors were generated. For in vitro experiments in human astrocyte cell lines, we used AAVs expressing wild-type HTT (25Q) or mutant HTT (103Q) under the control of a ubiquitous CMV promoter. These vectors are referred to as AAV-HTT(25Q) and AAV-mHTT(103Q). For in vivo experiments modeling non-cell autonomous pathology, AAVs expressing the same HTT constructs were driven by the neuron-specific human Synapsin (hSyn) promoter. Both sets of HTT-expressing AAVs were packaged with the AAV-DJ serotype. The astrocyte-specific WNT5B overexpression vector (AAV-GFAP-WNT5B) utilizes the human GFAP (GfaABC1D) promoter and is packaged with the AAV2/5 serotype to increase astrocyte transduction efficiency.^68–71^ For the gene silencing experiments, a Cre-dependent system in which an astrocyte-specific Cre-expressing virus (AAV-GFAP-Cre) was coinjected with our custom-generated Cre-on shRNA vectors for *Wnt5b*, *Nfatc2*, and *Mmp14 was used*. High-titer AAVs were produced by transiently transfecting HEK293T cells with the desired AAV plasmid, the corresponding serotype-specific packaging plasmid (pRC-DJ or pRC5), and a pHelper plasmid. After 72 h of incubation, the cell lysates were treated with benzonase (50 units/ml, Sigma, USA) to degrade residual nucleic acids. The viral particles were then purified and concentrated via a heparin column (GE Healthcare, Sweden) combined with a 100 kDa molecular weight cutoff filtration tube (Millipore, USA). Viral titers were quantified via qPCR.

### Behavioral tests

**Rotarod test:** The rotarod test was performed weekly on N171-82Q, N171-82Q + WNT5B, and littermate WT control mice following a previously described method.^72^ **Cylinder test:** Spontaneous movement was assessed by placing the mice in a small transparent cylinder (height: 15.5 cm, diameter: 12.7 cm), as described by Lee et al.^67^. The spontaneous activity of the mice was videotaped for 3 minutes. The video recordings were then reviewed in slow motion by an experimenter who was blinded to the mouse genotype. Rearing was recorded whenever a mouse made a vertical movement with both forelimbs off the ground. **Tail Suspension Test:** Limb movement and dystonia were assessed via the tail suspension test as previously described.^67^ The mice were videotaped in a ventral posture while being suspended by their tails for 10 seconds, followed by touchdown and an additional suspension for 20 seconds (total suspension time of 30 seconds). The numbers of forelimb and hindlimb clasps were counted through slow-motion video analysis. **Computer-assisted gait analysis test:** Mice were placed inside a wheel, and the rotation speed was gradually increased from 4 to 15 rpm over 3 minutes. The test was recorded from the bottom and front using two cameras immediately after the wheel-running training phase. For footprint analysis, the hindlimbs and forelimbs of the mice were marked with different colors via a nontoxic animal marking stick (MS Schippers, AH Bladel) 4 h before the test.^73^ Gait analysis was conducted by detecting the colored hindlimbs with EthoVision XT software (Noldus, version 13, USA). Seven strides with continuous ambulatory movement were analyzed for each mouse.

### Preparation of RNA libraries for sequencing

Total RNA libraries were constructed using the NEBNext Ultra RNA Library Prep Kit (#E7530, NEBNext, USA) together with NEBNext Multiplex Oligos for Illumina (#E7335, USA) and the poly(A) mRNA Magnetic Isolation Module (#E74900, USA), following the supplier’s recommended workflow. A detailed protocol is publicly accessible on the manufacturer’s website (https://international.neb.com/products/e7530-nebnext-ultra-rna-library-prep-kit-for-illumina#Product%20Information). Library concentration and fragment integrity were validated using an Agilent 2100 Bioanalyzer equipped with a High-Sensitivity DNA Kit (Agilent Technologies, USA). For sequencing, libraries were processed according to the *HiSeq Reagent Kit Preparation Guide* (Illumina, USA), consistent with previously reported methods.^74^ The pooled library was adjusted to 2 nM, denatured using 0.2 N NaOH, and subsequently diluted to 20 pM with Illumina HT1 buffer prior to loading. A total volume of 600 µl of the prepared library—along with read 1, read 2, and index primers—was applied to a 150-cycle (2 × 75 bp paired-end) HiSeq reagent cartridge (Illumina, USA) and sequenced on a HiSeq high-throughput platform. Following completion of the paired-end run, base calling was performed, and barcode-matched reads were demultiplexed to generate FASTQ files for downstream analysis.

### RNA-seq analysis

Adapter sequences and bases with quality scores below 20 were trimmed via Cutadapt.^75^ The reads were mapped to the mouse reference genome (GRCm38, mm10) via STAR (version 2.7.0) with the default settings.^76^ Duplicate reads at identical genomic locations were removed via Picard, and reads with MAPQ < 5 were excluded. HTSeq was employed to count reads within exons for each gene,^77^ excluding genes with zero counts across all samples. Initially, genes with read counts above 5 were considered expressed. Differentially expressed genes (DEGs) were then identified from these expressed genes with a fold change > 2 and P value < 0.05 via DESeq.^78^

### Western blot analysis

The membranes were blocked in 5% skim milk in TBST (Tris, pH 7.4; 150 μm NaCl; 0.05% Tween 20) for 1 hour at room temperature. The blots were incubated with the following primary antibodies at 4 °C overnight: anti-WNT5B (1:100, Santa Cruz, USA), anti-NFATC2 (1:1000, Invitrogen, USA), anti-MMP14 (1:2000, Abcam, USA), anti-DARPP32 (1:1000, Abcam, USA), anti-GAPDH (1:5000, Bioworld, USA), anti-ACTB/β-actin (1:10000, Santa Cruz, USA), and anti-TUBB3/tubulin (1:500, Merck, USA). After three washes with Tris-buffered saline containing 0.05% Tween 20, the blots were incubated at room temperature for 1 hour with horseradish peroxidase (HRP)-conjugated secondary antibodies (anti-rabbit HRP, Amersham Pharmacia, USA). Immunoreactive bands were developed via Immobilon Western ECL solution (Merck Millipore, USA) and visualized with an Image Station 4000MM (#745,280; Kodak, Japan). ACTB or TUBB3 served as the loading control. The original blots are presented in **Full blots file**.

### Immunoprecipitation (IP)

HEK293TN cells were transfected with pCMX-VP16-ERα and pBIND-NFATC2 full-length or deletion constructs for 48 h. The cells were harvested and lysed as described previously. The lysates were incubated with an anti-GAL4 antibody (7 µg/sample, Santa Cruz, USA) for 2 h at 4 °C, followed by the addition of protein A/G magnetic beads. The samples were incubated overnight at 4 °C with gentle rotation. After incubation, the beads were washed three times with lysis buffer to prevent nonspecific binding. Bound proteins were eluted by boiling the samples in 2X sample buffer. The eluted proteins were subjected to Western blot analysis. The blots were incubated overnight at 4 °C with an anti-ERα antibody (1:1000, GeneTex, USA) to detect the immunoprecipitated proteins.

### MMP14 promoter cloning and luciferase promoter assay

To investigate the transcriptional activity of the human *MMP14* promoter (Gene ID: 4323), a series of deletion constructs (100-bp, 200-bp, 300-bp, 500-bp, 1000-bp, and 1500-bp upstream regions, spanning from -100, -200, -300, -500, -1000, and -1500 to -1 relative to the translation start site) were generated via PCR using human astrocyte genomic DNA as the template. The primers used for amplification are listed in Supplementary Table 2. The PCR products were initially cloned and inserted into a TA vector via the TOPcloner™ TA Core Kit (Enzynomics, Korea) and verified by sequencing (Macrogen, Korea). The confirmed sequences were then excised via the *BglII* and *Acc65I* restriction enzymes and subcloned and inserted into the *pGL4.14-Enhancer-Luc* vector (Promega, USA) at the corresponding sites. Additionally, a deletion construct spanning from -200 to -90 bp was generated from the 200-bp promoter fragment via the EZchange™ Site-Directed Mutagenesis Kit (Enzynomics, Korea) with specific primers (forward: 5’-AGATCTGGCCTCGGCGGC-3’ and reverse: 5’-GACGTGGTTGTTTTAGCC-3’). The reporter plasmids were cotransfected with the *NFATC2* expression plasmid into human astrocytes via Lipofectamine™ 3000 (Thermo Fisher). After 24 h, the cells were washed with PBS, lysed with 5X lysis reagent, and detected via the luciferase assay buffer provided in the Luciferase Assay System (Promega, USA).

### Statistical analysis

All quantitative values are expressed as mean ± SEM. Behavioral tracking was conducted using EthoVision XT (Noldus, USA). Image processing and three-dimensional reconstruction were performed using ImageJ (NIH, USA) and IMARIS (Oxford Instruments, UK), respectively. Statistical tests were carried out using GraphPad Prism v8.4.3 (GraphPad Software, USA). For comparisons between two independent groups, an unpaired two-tailed Student’s t-test was applied, whereas repeated-measures ANOVA was used to analyze datasets involving more than two groups. When dealing with nested datasets or repeated observations, a linear mixed-effects model (LMM) was implemented to assess group differences. tatistical significance was defined as follows: * p < 0.05, ** p < 0.01, and *** p < 0.001. Sample sizes were not predetermined using statistical power calculations but were selected based on standards and precedents from prior literature.^66,67^

## Supplementary information

Full blots

Supplementary Materials for Astrocytic noncanonical WNT5B signaling modulates extracellular matrix remodeling and neuropathology in Huntington’s disease

## Data Availability

The RNA sequencing data generated in this study were deposited in the GEO database (accession number: GSE64810). All supporting information and data are available in the article and supplementary files.
