# Supplementary figures and images for: Astrocytic noncanonical WNT5B signaling modulates extracellular matrix remodeling and neuropathology in Huntington’s disease
Source: Signal Transduct Target Ther. 2026 Jan 19;11:23. doi: 10.1038/s41392-025-02545-9 (PMC12812802; doi:10.1038/s41392-025-02545-9)

**
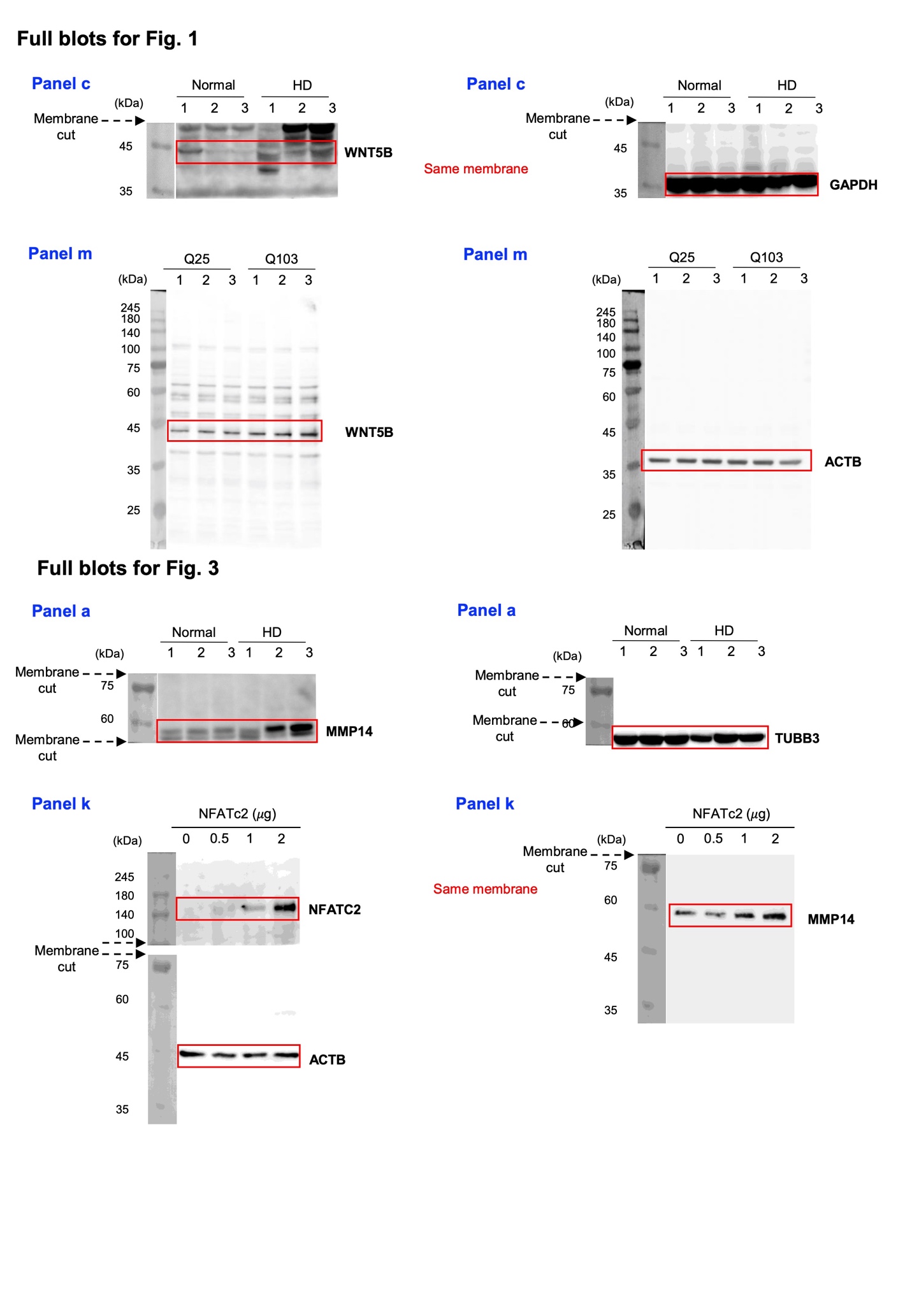
**

**
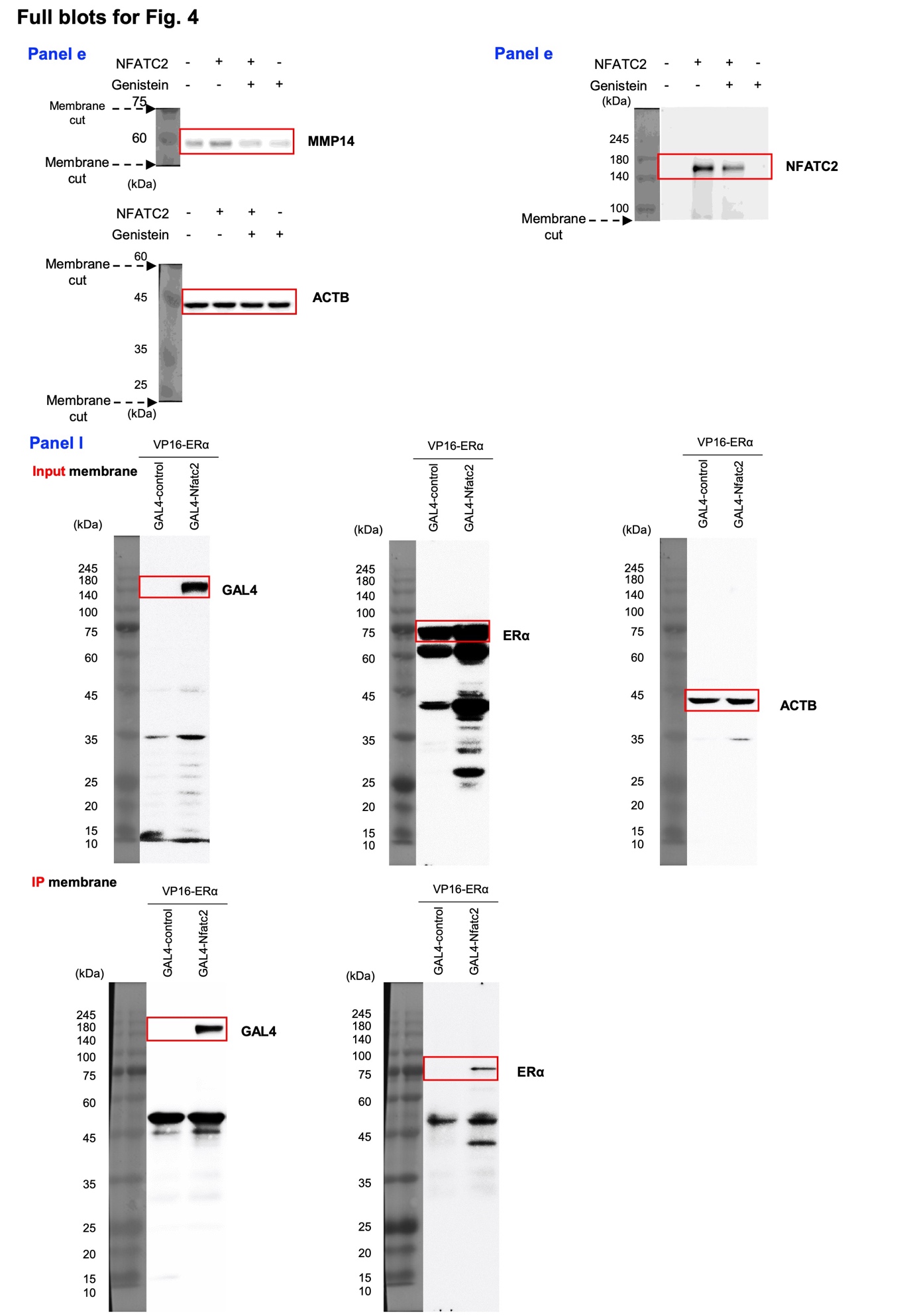
**

**
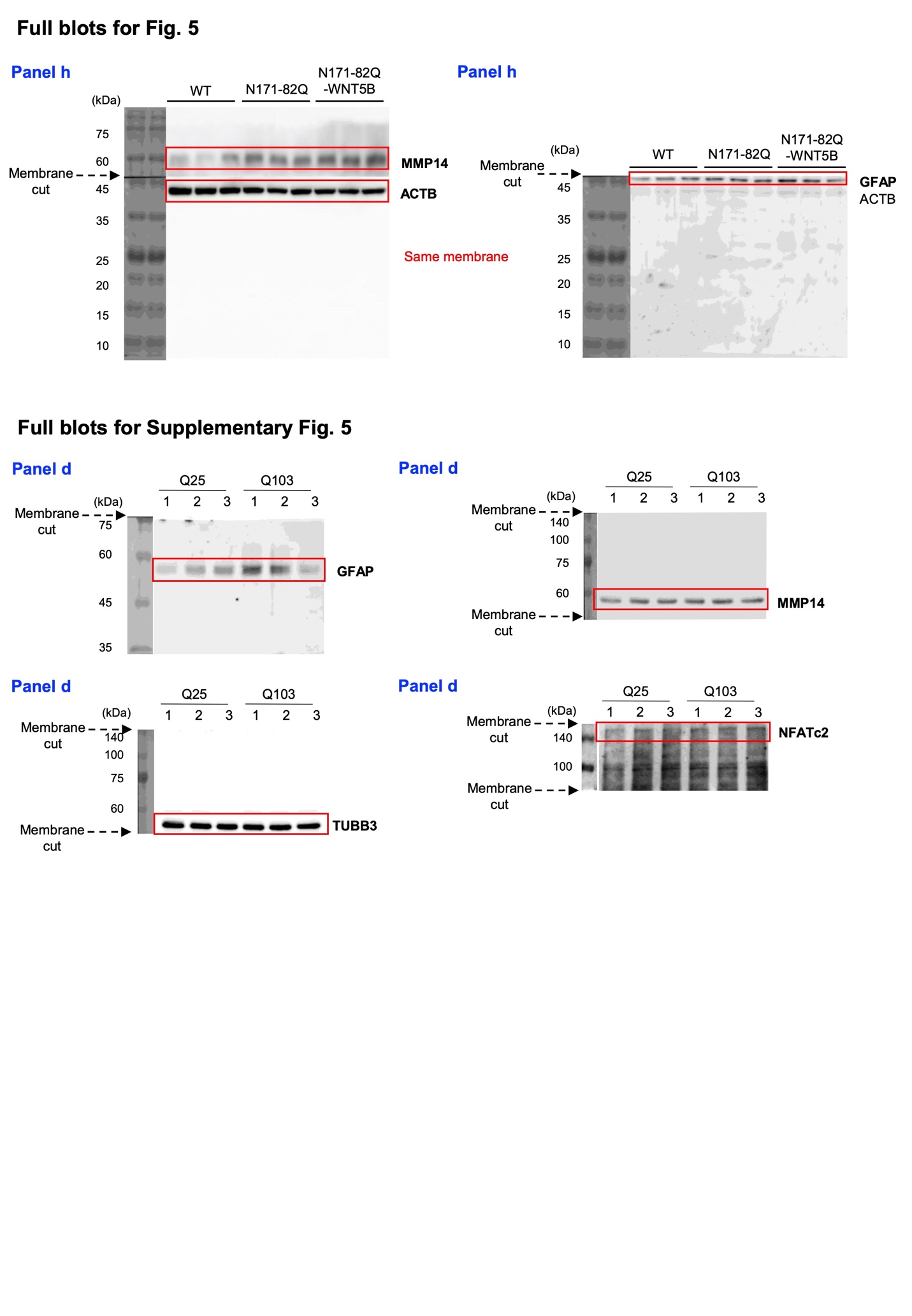
**

**
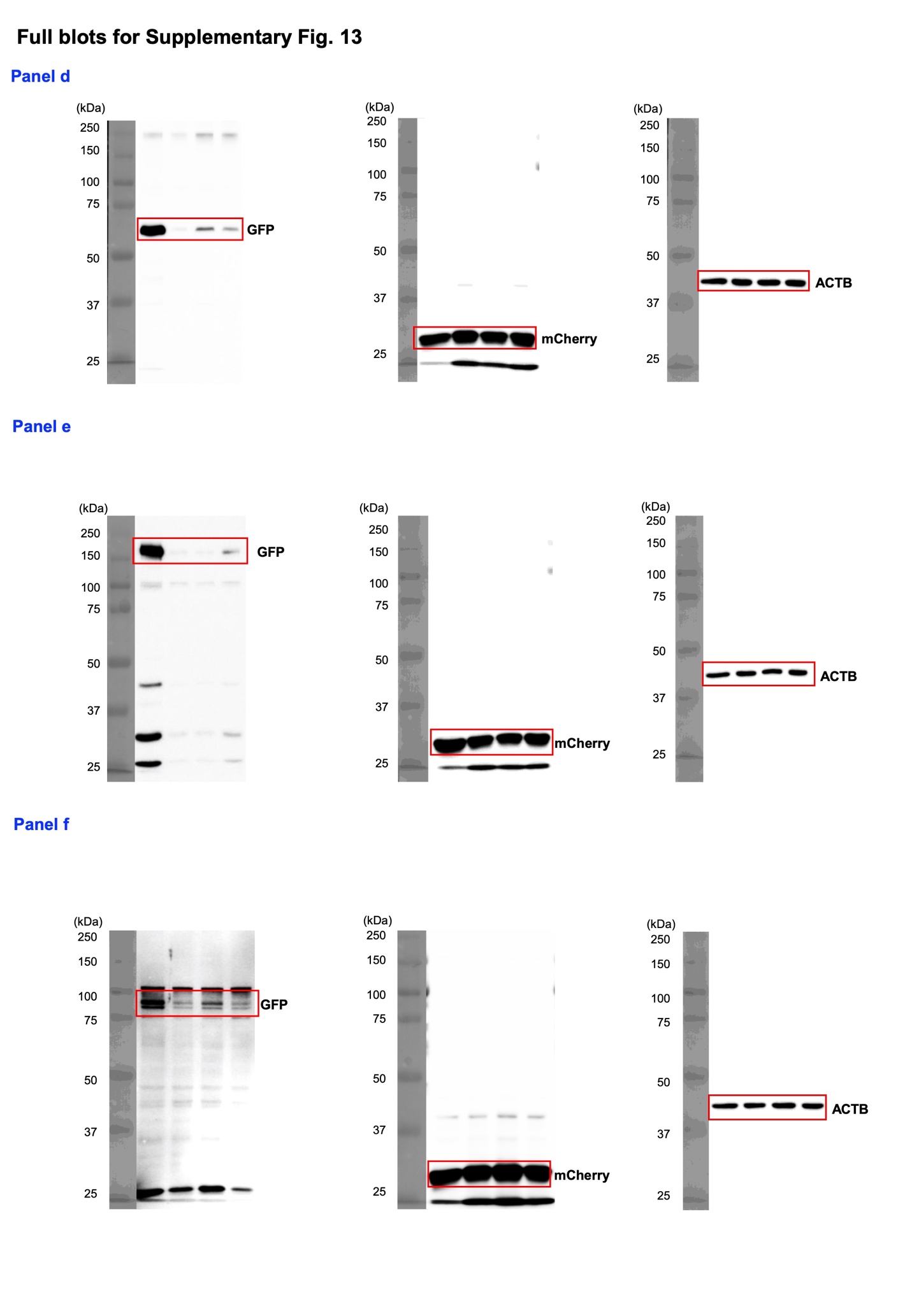
**

Supplement: Supplementary file 1 — Full blots [file 41392_2025_2545_MOESM1_ESM.docx]
