# Supplementary material for: Astrocytic noncanonical WNT5B signaling modulates extracellular matrix remodeling and neuropathology in Huntington’s disease
Source: Signal Transduct Target Ther. 2026 Jan 19;11:23. doi: 10.1038/s41392-025-02545-9 (PMC12812802; doi:10.1038/s41392-025-02545-9)
Supplement: Supplementary file 2 — Supplementary Materials for Astrocytic noncanonical WNT5B signaling modulates extracellular matrix remodeling and neuropathology in Huntington’s disease [file 41392_2025_2545_MOESM2_ESM.docx]

Supplementary Materials for

**Astrocytic noncanonical WNT5B signaling modulates**

**extracellular matrix remodeling and neuropathology**

**in Huntington’s disease**

Phuong Thi Thanh Nguyen^#^, Ali Yousefian-Jazi^#§^, Seung Jae Hyeon^#^, Soomin Lee, Seung Chan Kim, Uiyeol Park, Yeeun Jeong, Sojung Kim, Suhyun Kim, Yeyun Kim, Hannah L. Ryu, Kyung Eun Lee, Thor D. Stein, Richard H. Myers, Eun Mi Hwang,

Junghee Lee^*^, and Hoon Ryu^*^

Correspondence to: Junghee Lee [junghee@bu.edu](mailto:junghee@bu.edu); Hoon Ryu [hoonryu@kist.re.kr](file:///D:\Phuong\Projects\Genistein-HD\Revision\final%20revision\hoonryu@kist.re.kr)

**This PDF file includes:**

Figures S1 to S15

Tables S1 to S4

References


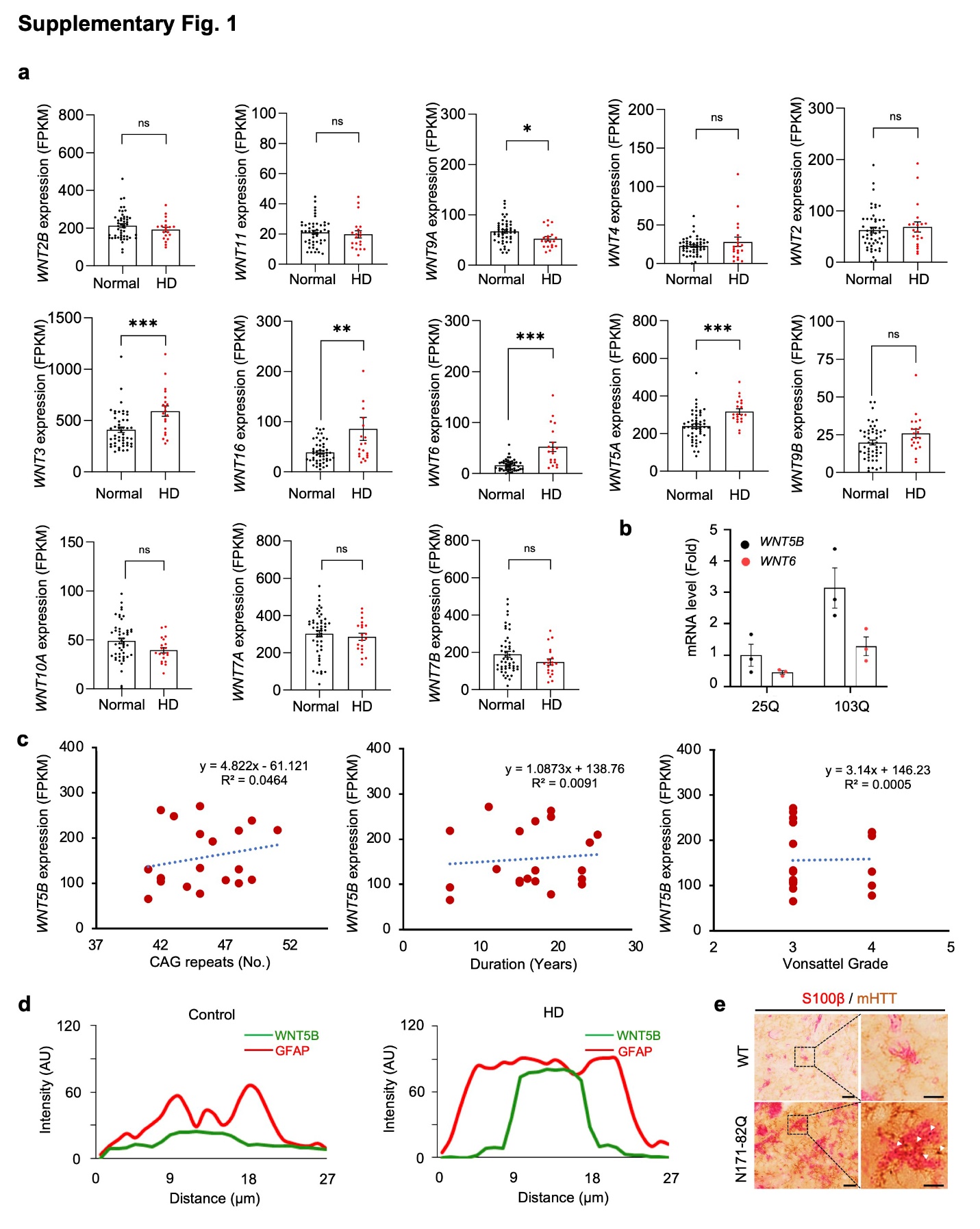


Figure. S1. *WNT* family expression levels in HD.

(a) RNA-seq transcriptome data showing *WNT* gene family expression levels (FPKM) in the prefrontal cortex of postmortem samples from normal subjects (*N* = 49) and HD patients (*N* = 20). Student's t-test (*, *p* < 0.05; **, *p* < 0.01; ***, *p* < 0.001). (b) Relative mRNA levels of *Wnt5b* and *Wnt6* were measured by RT-qPCR in human astrocytes transduced with AAV expressing wild-type *HTT* (25Q) or mutant *HTT* (103Q). (c) Correlation analysis between CAG repeat length (X-axis) and *WNT5B* expression (Y-axis, FPKM) in HD patient samples (*N* = 20). Linear regression analysis shows a weak positive trend. (d) Representative colocalization histograms of WNT5B and GFAP immunofluorescence signal in the striatal tissue of control (Normal) and HD patient. This data was derived from main Fig. 1 panel E. (e) Double chromogenic immunostaining images with anti-S100β (red) and mHTT aggregates (brown) in the striatum of WT and N171-82Q mice. Insets highlight that mHTT aggregates (white arrowheads) are found in S100β-positive astrocytes in the striatum of N171-82Q mice. Scale bars: 20 μm.


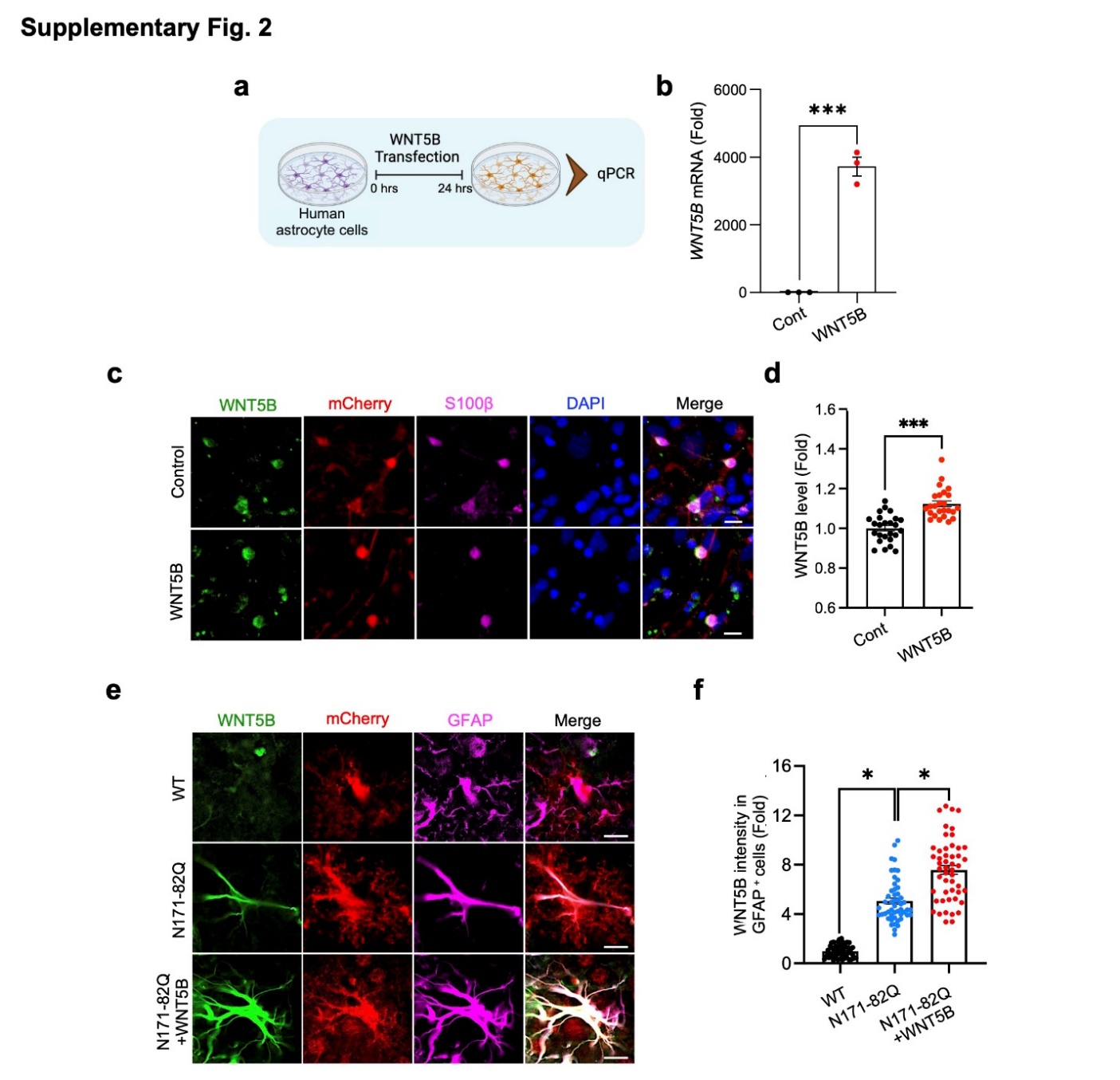


Figure. S2. Verification of *WNT5B* overexpression in cells and mouse models.

(a) Experimental design for *WNT5B* transfection into human astrocytes for 24 hours, followed by mRNA expression analsis using qPCR. (b) WNT5B overexpression increased *WNT5B* mRNA levels in human astrocytes. Student’s t-test (***, *p*< 0.001). (c) Immunofluorescence staining of primary striatal astrocytes transduced with AAV-GFAP (pro)-mCherry or AAV-GFAP (pro)-WNT5B-mCherry virus. The merged panels show an increased colocalization of WNT5B with astrocyte markers. The nuclei were counterstained with DAPI. Scale bar: 20 µm. (d) Quantification analysis of WNT5B intensity in S100β-positive cells. A total of 24 cells per group were counted (8 cells per well; 3 wells per group). Student’s t-test (***, *p* < 0.001). Error bars indicate mean ± SEM. (e) Immunostaining with anti-WNT5B, anti-mCherry, and anti-GFAP antibodies in the striatum from three groups of mice (WT, N171-82Q, and N171-82Q + WNT5B). Scale bars: 10 µm. (f) Quantification is of WNT5B intensity in mCherry and S100β-positive cells. A total of 50 cells per group were counted (10 cells per mouse; *N* = 5 mice per group). One-way ANOVA (*, *p* < 0.05).


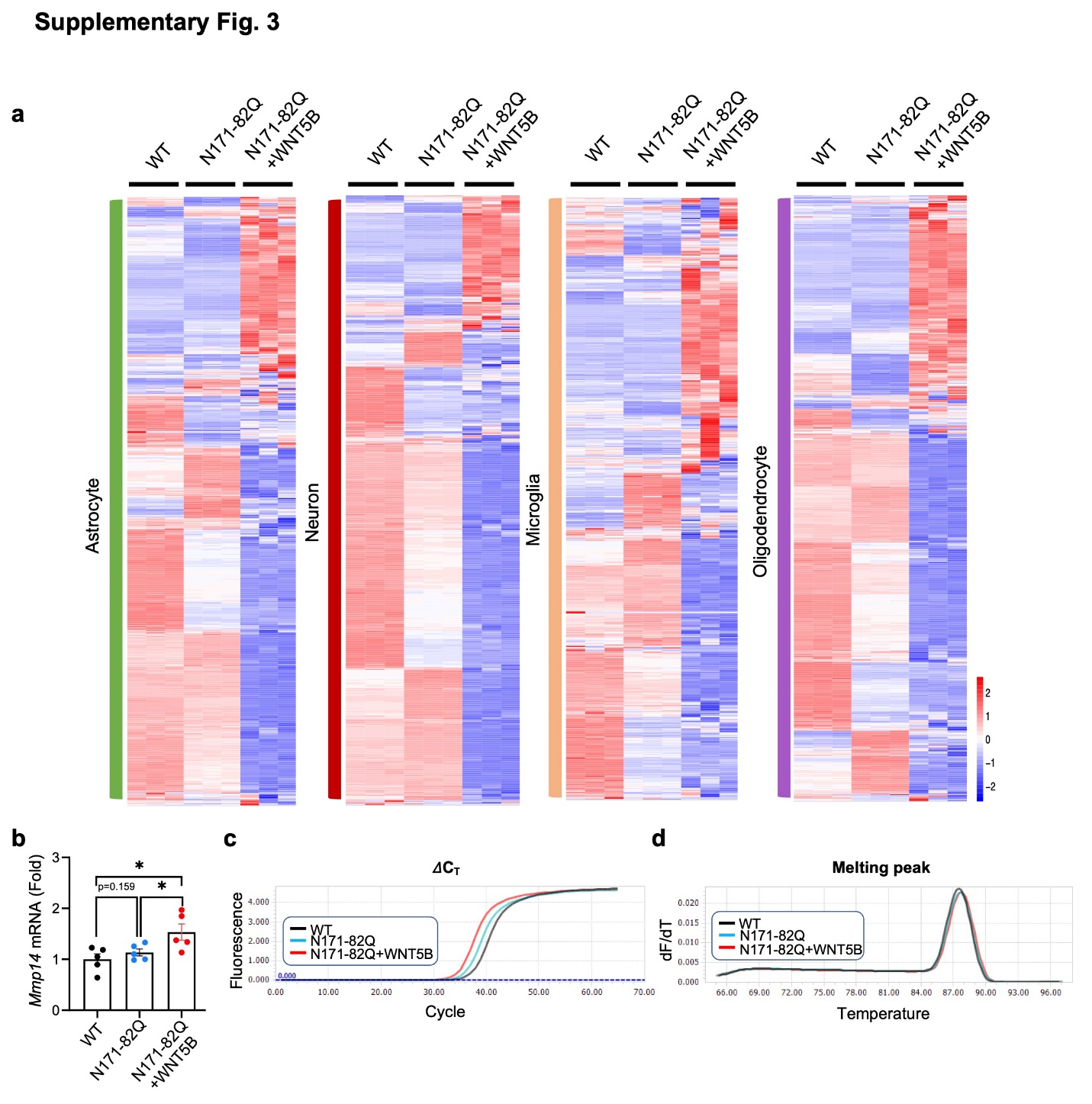


Figure. S3. *WNT5B* overexpression alters cell-type-specific transcriptome signatures and elevates *Mmp14* expression in HD mice.

(a) Heatmaps show astrocytes-, neurons-, microglia-, and oligodendrocyte-specific genes expression level in striatum of WT (*N* = 3), N171-82Q (*N* = 3), and N171-82Q + WNT5B mice (*N* = 3). Data were obtained from bulk RNA-sequencing analysis, and clustered cell-type-specific transcriptome signatures using hierarchical methods with deconvolution algorism. (b) qRT-PCR validation of *Mmp14* mRNA expression in the dorsal striatal tissues from WT (*N* = 5), N171-82Q (*N* = 5), and N171-82Q + WNT5B mice (*N* = 5). Error bars represent mean ± SEM. (c) Amplification curves depicting ΔCᴛ values of *Mmp14* expression across the three experimental groups. (d) Melting curve analysis confirming the specificity of the qRT-PCR amplification.


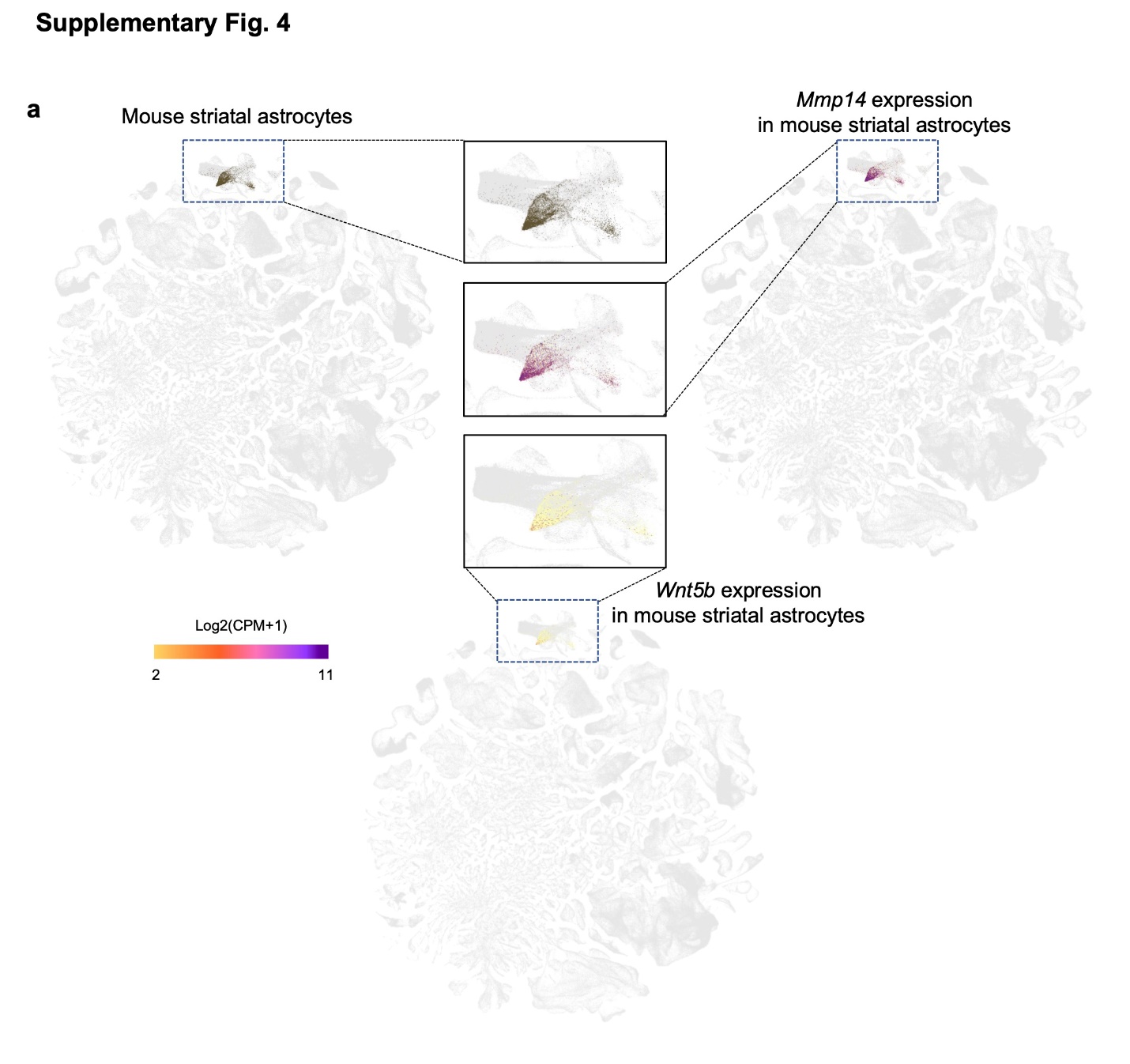


Figure. S4. *Mmp14* and *Wnt5b* are highly expressed in mouse striatal astrocytes. Scatterplots showing predominant expression of *Mmp14* and *Wnt5b* in mouse striatal astrocytes, based on 10x single-cell RNA sequencing of the whole mouse brain. The data were extracted from the Allen Institute Atlas (Yao, van Velthoven *et al.* 2023).


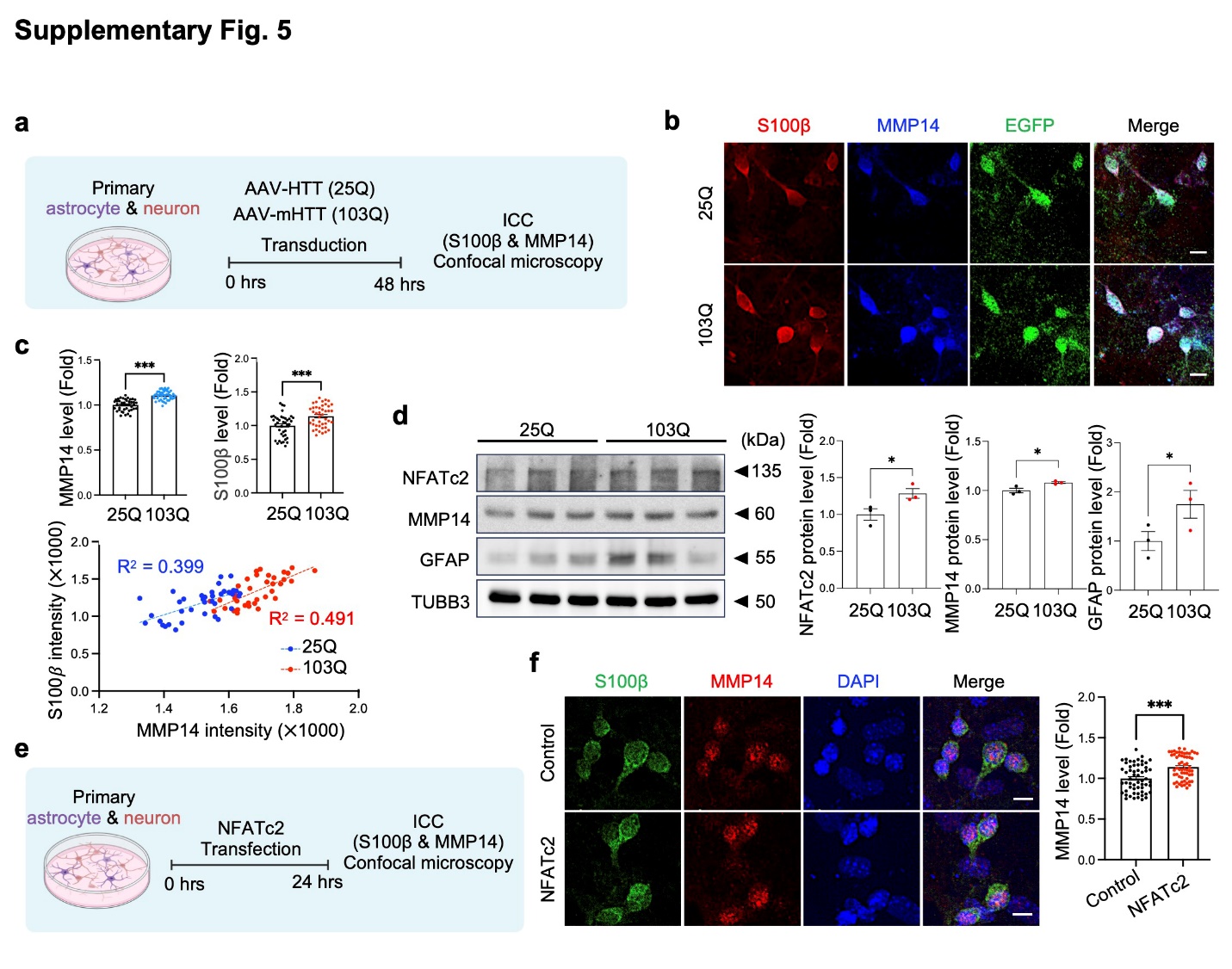


Figure. S5. Overexpression of *mHTT* and *NFATc2* induces MMP14 in primary mouse astrocytes.

(a) Experimental design for *wtHTT* and *mHTT* transient expression in primary astrocytes and neuron cultures, followed by ICC. The AAV-HTT (25Q)-EGFP or AAV-mHTT (103Q)-EGFP viruses were transduced in primary mouse astrocytes and neurons for 48 hours, followed by immunocytochemistry (ICC) with S100β (red) and MMP14 (blue). (b) Overexpression of mHTT (103Q) increased MMP14 and S100β immunoreactivities in the astrocytes. Scale bars: 20 μm. (c) Upper panels: Quantification of MMP14 and S100β levels in 25Q-EGFP and 103Q-EGFP virus transduced cells. Student’s *t*-test (***, *p* < 0.001). Bottom panel: Scatter plot represents positive correlation between S100β and MMP14 immunoreactivities. A total of 39 cells per group were counted (13 cells per well; 3 wells per group). (d) Western blot analysis for detecting NFATc2, MMP14, and GFAP protein levels from human astrocytes transfected by *wtHTT* (25Q) and *mHTT* (103Q). Right: Quantification of NFATc2, MMP14, and GFAP protein levels (3 samples per group). Error bars indicate mean ± SEM. *, *p* < 0.05. (e) Experimental design for *NFATc2* transfection in primary mouse astrocytes and neurons for 24 hours, followed by ICC. (f) *NFATc2* overexpression (OE) elevated MMP14 (red) immunoreactivity in striatal astrocytes. Scale bars: 20 μm. Right panel: quantification analysis of MMP14 levels in S100β-positive cells. A total of 60 cells per group were counted (20 cells per well; 3 wells per group). ***, *p* < 0.001.
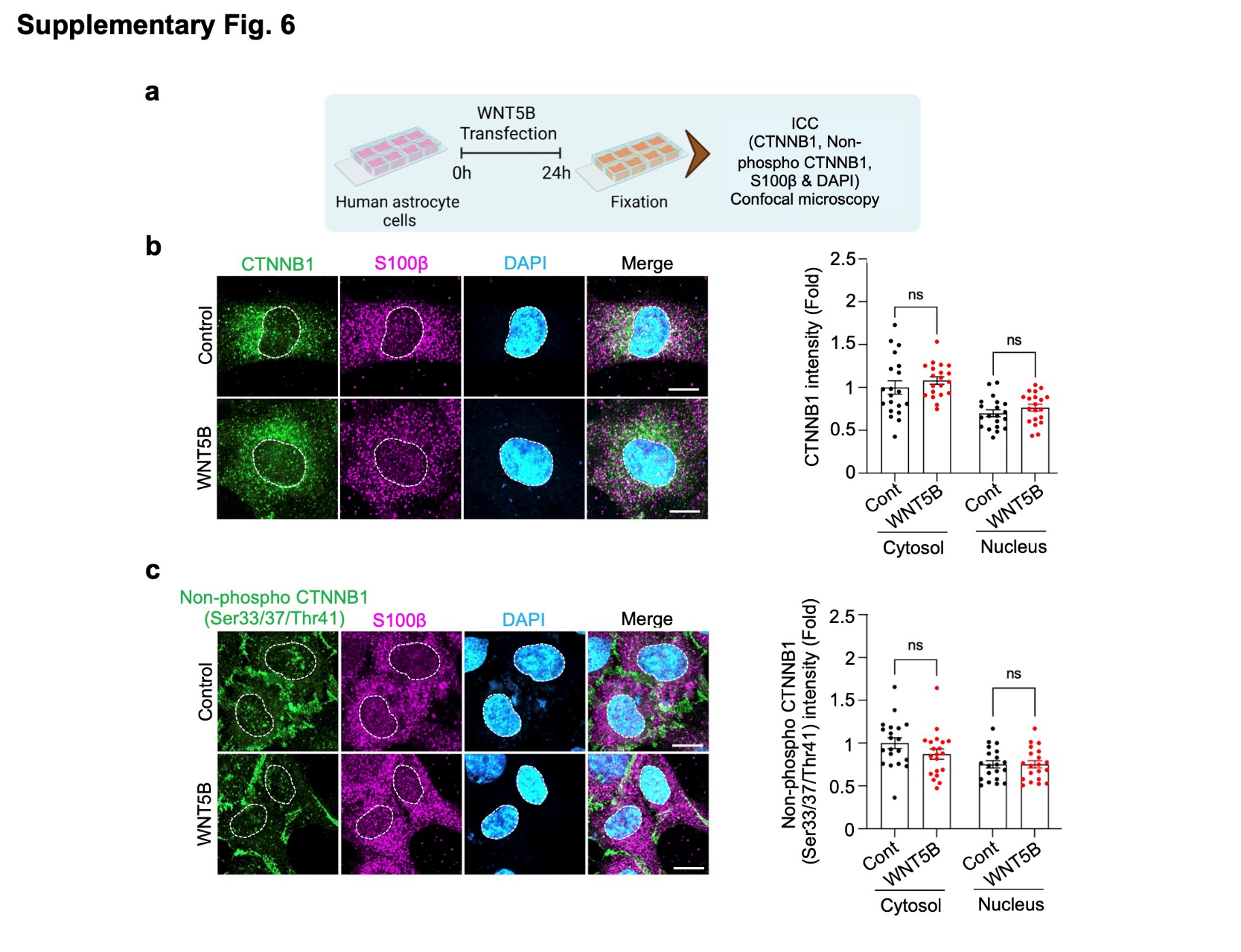


Figure. S6. *WNT5B* overexpression does not enhance active β-catenin nuclear translocation in human astrocytes.

(a) Experimental desing of *WNT5B* transient expression in human astrocyte cells followed by ICC. The pAAV-GFAP (pro)-mCherry or pAAV-GFAP (pro)-WNT5B-mCherry plasmids were transfected into human astrocyte cells for 24 hours, followed by ICC staining with anti-total β-catenin (CTNNB1) and active β-catenin (Non-phospho CTNNB1, Ser33/37/Thr41). (b) *WNT5B* overexpression did not alter total β-catenin protein levels in both the cytosol and the nucleus. Scale bar: 10 μm. Right: Quantification analysis of the total β-catenin intensity in cytosolic and nuclear compartments. A total of 30 cells per group were counted (10 cells per well; 3 wells per group). Error bars represents mean ± SEM. Student's *t*-test (ns, not significant). (c) *WNT5B* overexpression did not induce the nuclear translocation of active β-catenin (Non-phospho CTNNB1, Ser33/37/Thr41). Scale bar: 10 μm. Right panel shows quantification analysis of the intensity in cytosolic and nuclear compartments. A total of 30 cells per group were counted (10 cells per well; 3 wells per group). Student's *t*-test (ns, not significant).


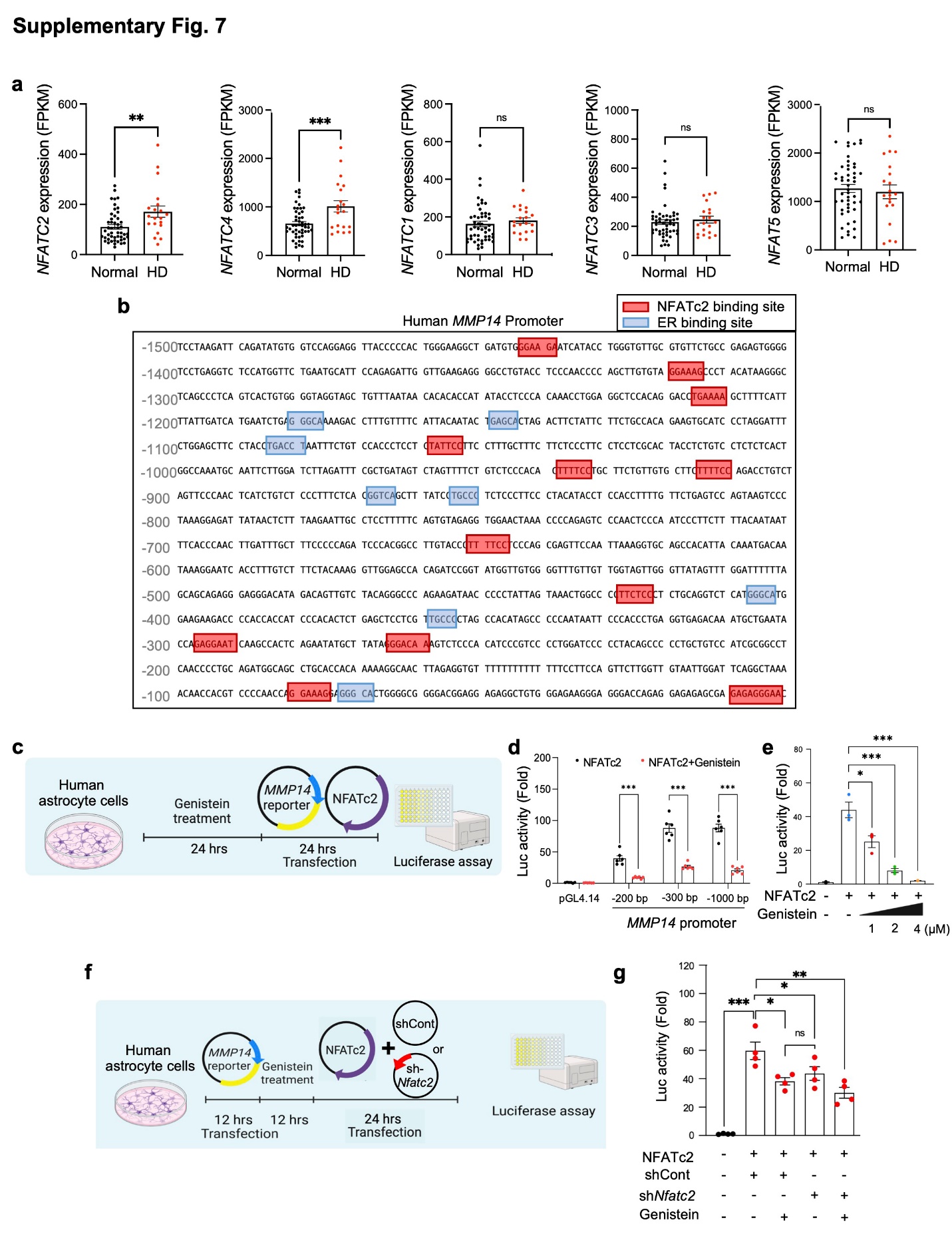


Figure. S7. NFATc2 activation in HD and its suppression of MMP14 transcriptional activity by phytoestrogen.

(a) RNA-seq analysis of *NFAT* family gene expression (FPKM) in the prefrontal cortex of control (Normal, *N* = 49) and HD (*N* = 20) postmortem samples. Bar plots indicate a significant increase in *NFATc2* and *NFATc4* expression in HD patients. (b) Predicted NFATc2 and ER binding sites in the sequence of the MMP14 promoter region from -1500 bp upstream of the transcription start site. NFATc2 binding sites are highlighted in red, and ER binding sites are highlighted in blue. Data extracted from TRANSFAC 6.0-based Patch 1.0 algorithm (Matys, Kel-Margoulis *et al.* 2006). (c) Experimental design for a luciferase assay in human astrocytes pretreated with genistein, followed by transfection with an MMP14 reporter and NFATc2. (d) Genistein reduced NFATc2-enhanced MMP14 promoter activity across various promoter lengths (-1000, -300, and -200 bp). Data represents the mean of six independent experiments. Error bars indicate mean ± SEM. One-way ANOVA (*, *p* < 0.05; **, *p* < 0.01; ***, *p* < 0.001). (e) Genistein decreased MMP14 promoter activity (-200 bp) in a dose-dependent manner. Data represent the mean of three independent experiments. *, *p* < 0.05; **, *p* < 0.01; ***, *p* < 0.001. (f) Experimental design for comparing the effects of genistein treatment and sh*Nfatc2* transfection on MMP14 promoter activity. (g) Both genistein and *Nfatc2* knockdown similarly reduced NFATc2-induced MMP14 reporter activity. Data represent the mean of four independent experiments. *, *p* < 0.05; **, *p* < 0.01; ***, *p* < 0.001.


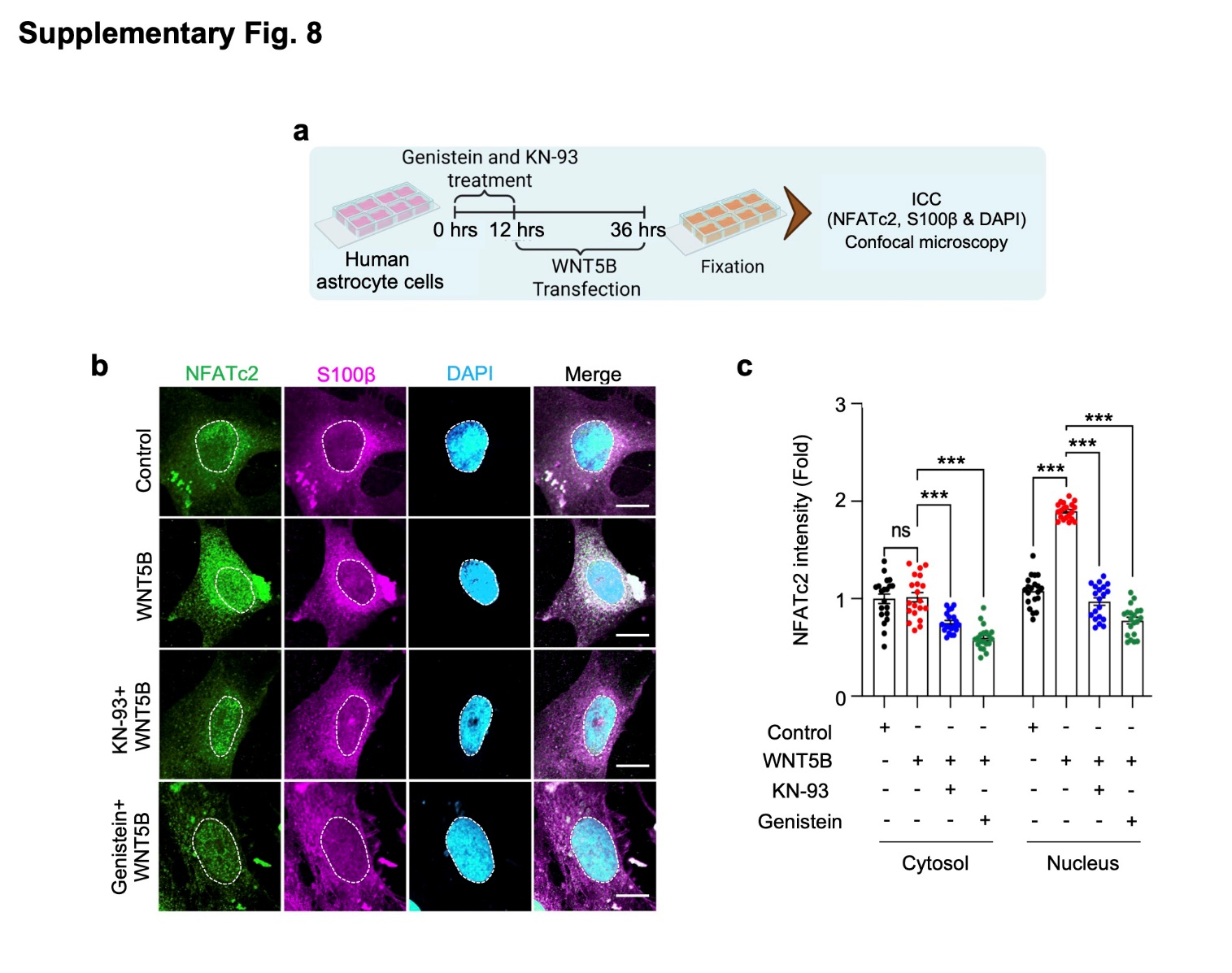


Figure. S8. WNT5B induces NFATc2 nuclear translocation via the WNT/Ca²⁺–CaMKII pathway, which is suppressed by KN-93 or genistein.

(a) Experimental design for pretreating human astrocytes with the CaMKII inhibitor KN-93 or genistein prior to transient WNT5B expression. (b) Pretreatment with KN-93 or genistein markedly attenuated WNT5B-induced NFATc2 nuclear translocation in human astrocytes. White dotted circles indicate the nucleus. Scale bars (white): 10 μm. (c) Quantification of NFATc2 fluorescence intensity in cytosolic and nuclear compartments. A total of 30 cells per group were analyzed (10 cells per well; 3 wells per group: Control, WNT5B, WNT5B + KN-93, WNT5B + Genistein). One-way ANOVA (***, *p* < 0.001).


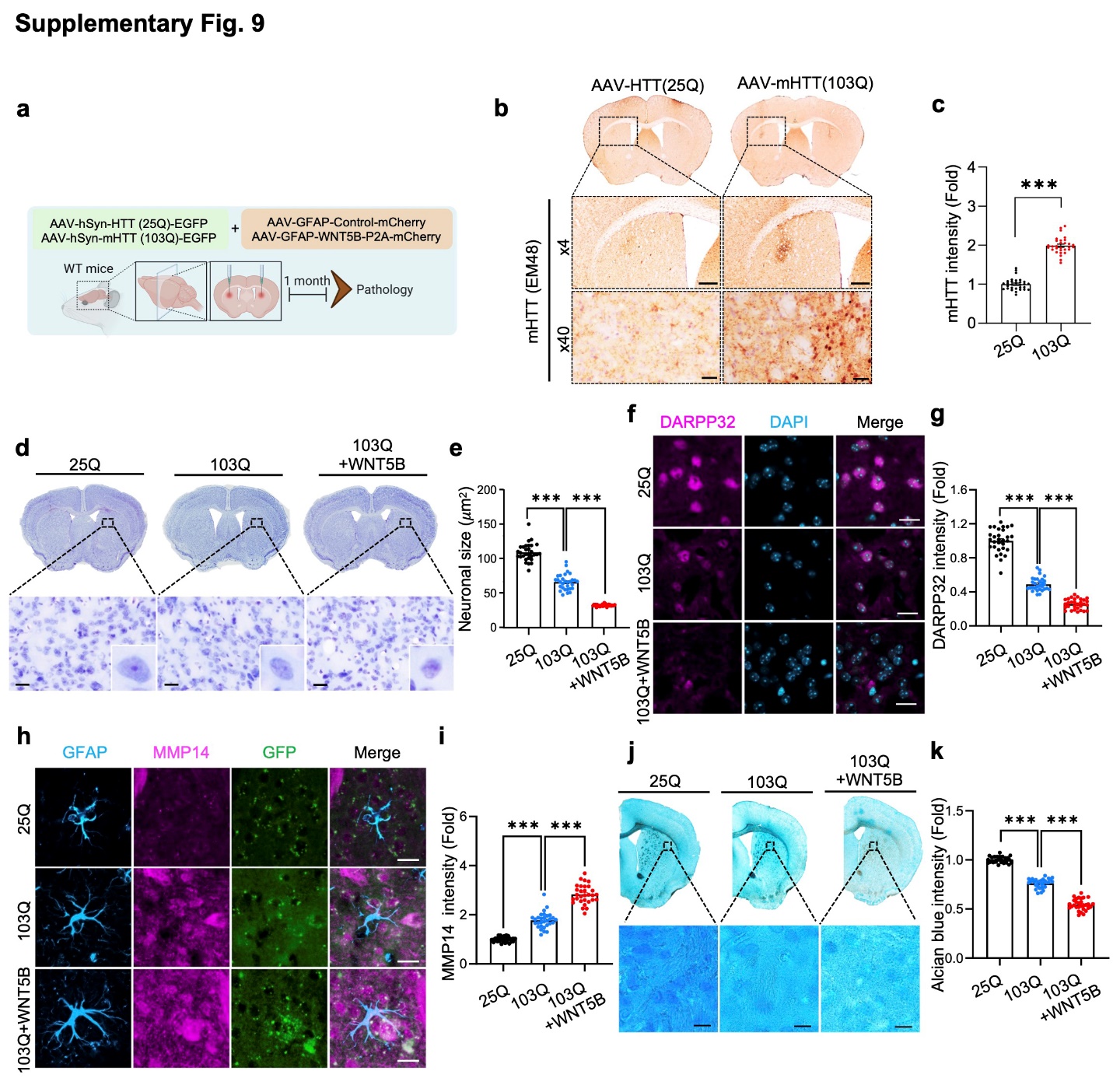


Figure. S9. WNT5B overexpression in the AAV-mHTT(103Q) model enhances pathology.

(a) Experimental design for the delivery of AAV-HTT (25Q) (control) or AAV-mHTT (103Q) (HD model) into the dorsal striatum of 25Q mice. For astrocyte-specific WNT5B overexpression, either pAAV-GFAP(pro)-Control-mCherry or pAAV-GFAP(pro)-WNT5B-P2A-mCherry was co-injected. Pathological analysis was performed three weeks post-injection. (b) mHTT (EM48) immunoreactivity was elevated in the striatum of AAV-mHTT (103Q) injected mice. Scale bars: 500 μm (10X); 100 μm (40X). (c) Quantification of mHTT intensity. A total of 30 cells per group were analyzed (10 cells per mouse; *N* = 3 mice per group). Student's *t*-test (*, *p* < 0.05; **, *p* < 0.01; ***, *p* < 0.001). (d) Representative images of cresyl violet staining. (e) Quantification of neuronal size in the dorsal striatum. A total of 30 cells per group were analyzed (10 cells per mouse; *N* = 3 mice per group). One-way ANOVA (*, *p* < 0.05; **, *p* < 0.01; ***, *p* < 0.001). (f) DARPP-32 immunoreactivity was decreased in the striatum of 103Q mice and further exacerbated in 103Q+WNT5B mice. The nuclei were counterstained with DAPI. Scale bars: 20μm. (g) Quantification of DARPP-32 intensity and cell number. A total of 30 cells per group were analyzed (10 cells per mouse; *N* = 3 mice per group). ***, *p* < 0.001. (h) Immunostaining showing MMP14 and GFAP levels. The nuclei were counterstained with DAPI. Scale bars: 20μm. (i) Quantification of MMP14 immunoreactivity in GFAP-positive cells. A total of 30 cells per group were analyzed (10 cells per mouse; *N* = 3 mice per group). ***, *p* < 0.001. (j) Alcian Blue staining showing glycosaminoglycan damage in 103Q + WNT5B mice. Scale bars: 20 μm. (k) Quantification of Alcian Blue density. Three ROIs (0.05 mm² each) per mouse were analysed (*N* = 3 mice per group). ***, *p* < 0.001).


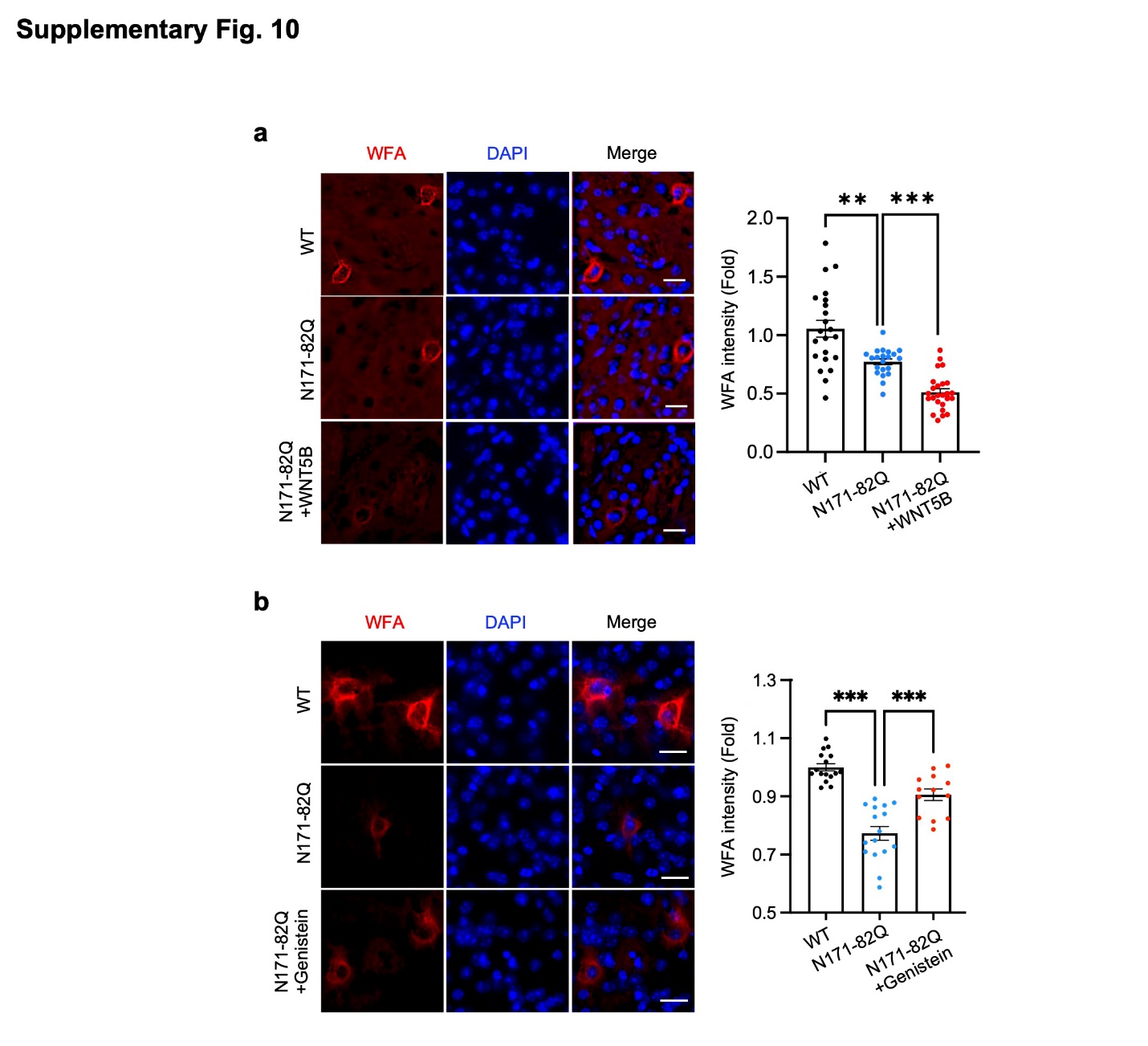


Figure. S10. WNT5B overexpression disrupts perineuronal nets (PNNs), while genistein treatment restores them in N171-82Q mice.

(a) WFA immunoreactivity was reduced in the striatum of N171-82Q + WNT5B mice. The nuclei were counterstained with DAPI. Scale bars (white): 20μm. Right: Quantification of WFA intensity. A total of 30 cells per group were counted (10 cells per mouse; *N* = 3 mice per group). Error bars indicate mean ± SEM. One-way ANOVA (*, *p* < 0.05; **, *p* < 0.01; ***, *p* < 0.001). (b) WFA immunoreactivity was elevated in the striatum of genistein-treated N171-82Q mice (N171-82Q + Genistein). The nuclei were counterstained with DAPI. Scale bars: 20μm. Right: Quantification of WFA intensity. A total of 15 cells per group were counted (5 cells per mous; *N* = 3 mice per group). ***, *p* < 0.001.


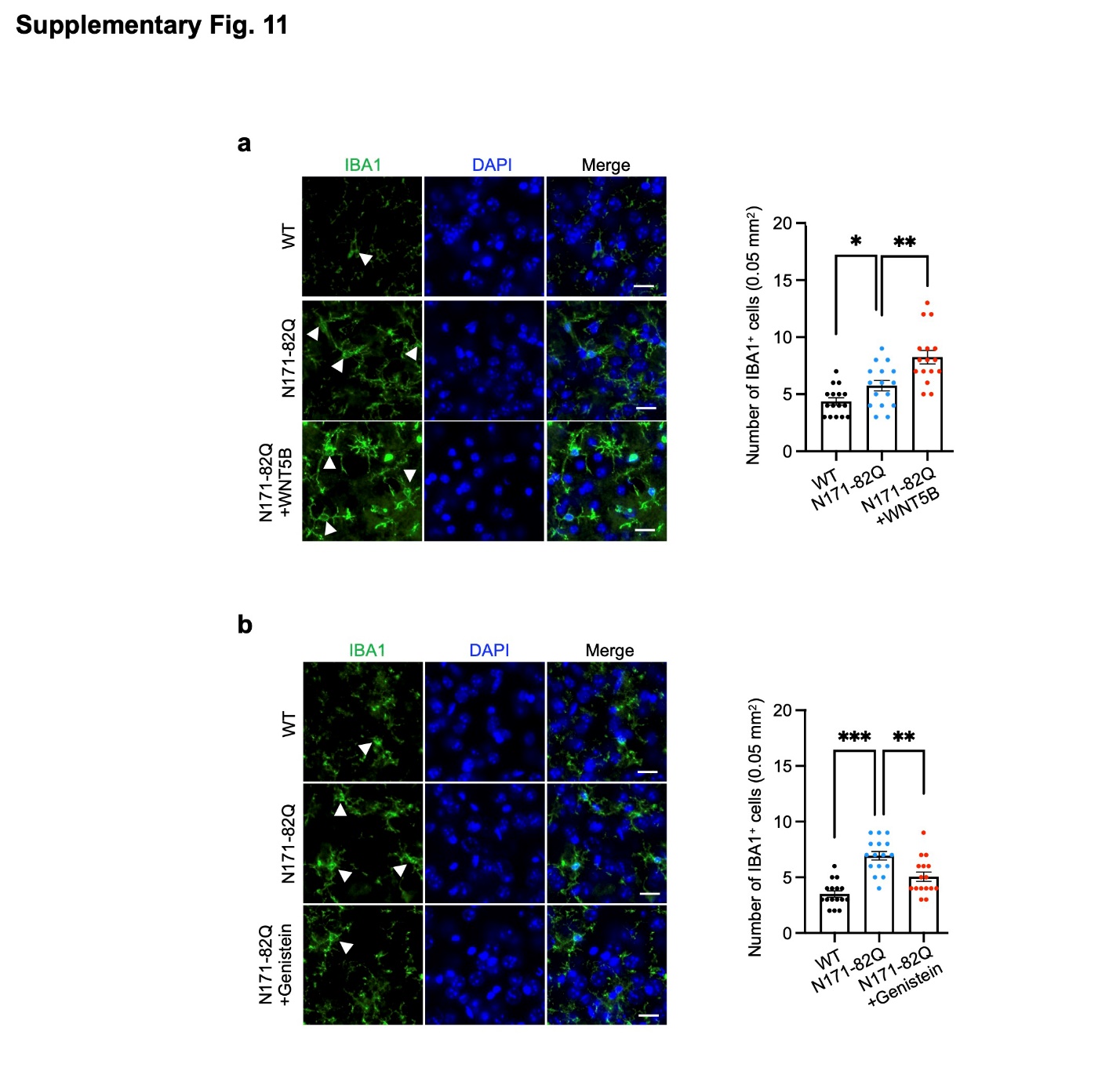


Figure. S11. WNT5B overexpression enhances microglial activation in the striatum of N171-82Q mice, which is attenuated by genistein treatment.

(a) IBA1 immunoreactivity was increased in the striatum of N171-82Q + WNT5B mice. Arrowheads (white) indicate IBA1-positive microglia. The nuclei were counterstained with DAPI. Scale bars: 20μm. Right: Quantification of the number of IBA1-positive microglia. Five ROIs (0.05 mm² each) per mouse were analyzed (*N* = 3 mice per group). Error bars indicate mean ± SEM. One-way ANOVA (*, *p* < 0.05; **, *p* < 0.01; **, *p* < 0.001). (b) IBA1 immunoreactivity was decreased in the striatum of genistein-treated N171-82Q mice. Arrowheads indicate IBA1-positive microglia. The nuclei were counterstained with DAPI. Scale bars: 20μm. Right: Quantification of the number of IBA1-positive microglia. Five ROIs (0.05 mm² each) per mouse were analyzed (*N* = 3 mice per group). **, *p* < 0.01; **, *p* < 0.001

**
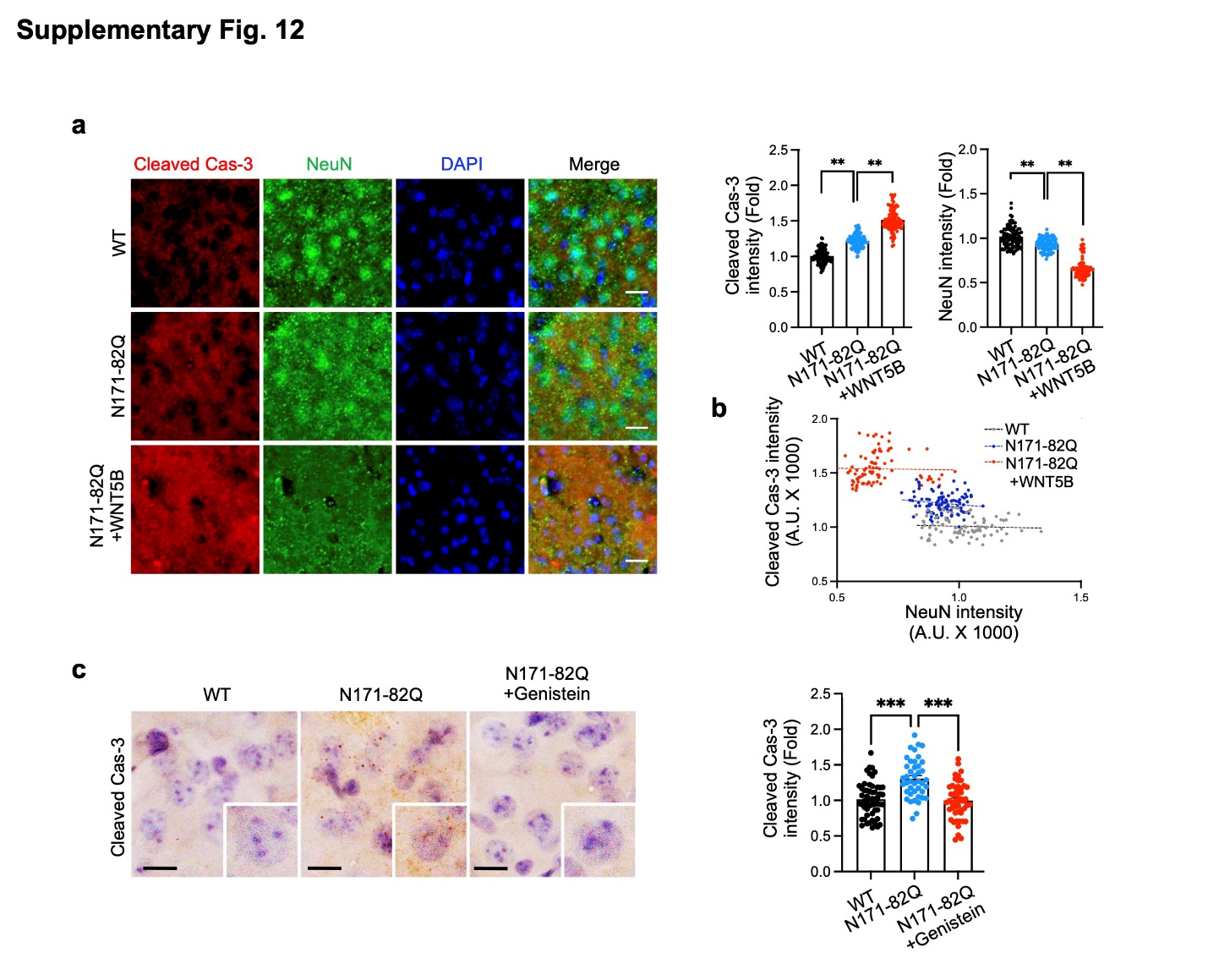
**

Figure. S12. WNT5B overexpression promotes neuronal apoptosis, while genistein treatment reduces it in N171-82Q mice.

(a) Cleaved Caspase-3 immunoreactivity was increased in the striatum of N171-82Q+WNT5B mice. The nuclei were stained with DAPI. Scale bars (white): 20 μm. Right panel shows quantification of cleaved Caspase-3 and NeuN intensities. A total of 30 cells per group were counted (10 cells per mouse; *N* = 3 mice per group). One-way ANOVA (*, *p* < 0.05; **, *p* < 0.01; ***, *p* < 0.001). (b) Scatter plot showing the correlation between Cleaved Caspase-3 and NeuN immunoreactivity levels in striatal neurons. (c) Cleaved Caspase-3 immunoreactivity was decreased in the striatum of genistein-treated N171-82Q mice. Arrowheads indicate cleaved Caspase-3-positive cells. ). The nuclei were stained with hematoxylin. Scale bars: 10 μm. Right: Quantification of cleaved Caspase-3 intensity. A total of 48 cells per group were counted (16 cells per mouse; *N* = 3 mice per group. One-way ANOVA (*, *p* < 0.05; **, *p* < 0.01; ***, *p* < 0.001).


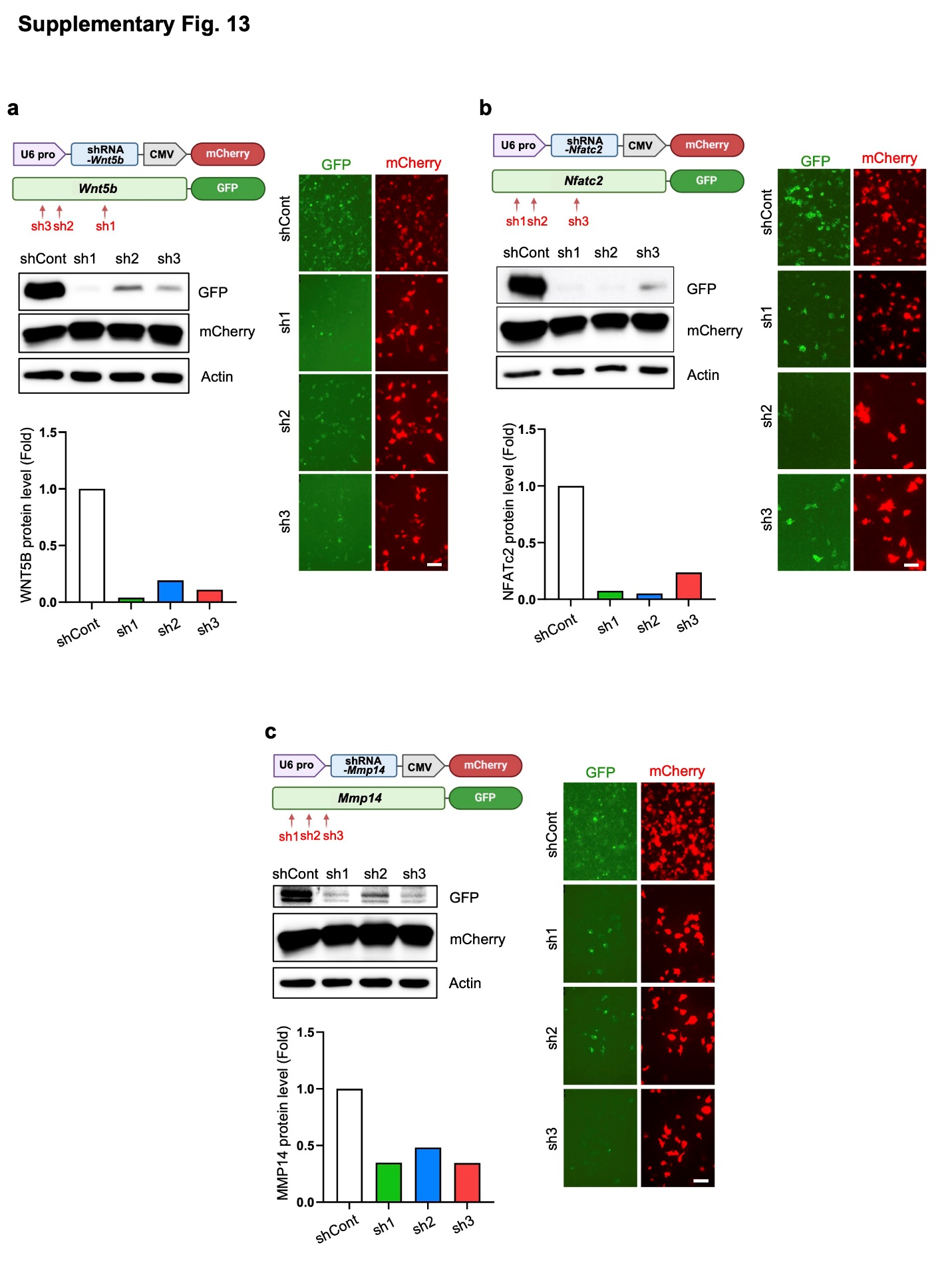


Figure. S13. Verification of *Wnt5b*, *Nfatc2*, and *Mmp14* shRNA efficiency.

(a) Validation of shRNA construct targeting *Wnt5b*. Schematic illustration of three shRNA constructs (sh1, sh2, sh3) and their targeting sites within the *Wnt5b*-GFP transcript. WB analysis comparing WNT5B-GFP protein levels in HEK293T cells co-transfected with control shRNA (shCont), sh1, sh2, or sh3. Quantification of WNT5B-GFP expression normalized to shCont, confirming the knockdown efficiency of each construct. Representative fluorescence images of HEK293T cells co-transfected with Wnt5b-GFP and individual shRNA constructs (shCont, sh1, sh2, sh3) demonstrating differential GFP signal intensity corresponding to knockdown efficiency. (b) Validation of shRNA construct targeting *Nfatc2*. Schematic illustration of three shRNA constructs (sh1, sh2, sh3) and their targeting sites within the *Mmp14*-GFP transcript. WB analysis comparing NFATc2-GFP protein levels in HEK293T cells co-transfected with control shRNA (shCont), sh1, sh2, or sh3. Quantification of NFATc2-GFP expression normalized to shCont, confirming the knockdown efficiency of each construct. Representative fluorescence images of HEK293T cells co-transfected with *Nfatc2*-GFP and individual shRNA constructs (shCont, sh1, sh2, sh3) demonstrating differential GFP signal intensity corresponding to knockdown efficiency. (c) Validation of shRNA construct targeting *Mmp14*. Schematic illustration of three shRNA constructs (sh1, sh2, sh3) and their targeting sites within the *Mmp14*-GFP transcript. WB analysis comparing MMP14-GFP protein levels in HEK293T cells co-transfected with control shRNA (shCont), sh1, sh2, or sh3. Quantification of MMP14-GFP expression normalized to shCont, confirming the knockdown efficiency of each construct. Representative fluorescence images of HEK293T cells co-transfected with *Mmp14*-GFP and individual shRNA constructs (shCont, sh1, sh2, sh3) demonstrating differential GFP signal intensity corresponding to knockdown efficiency. Scale bars: 100 μm.

**
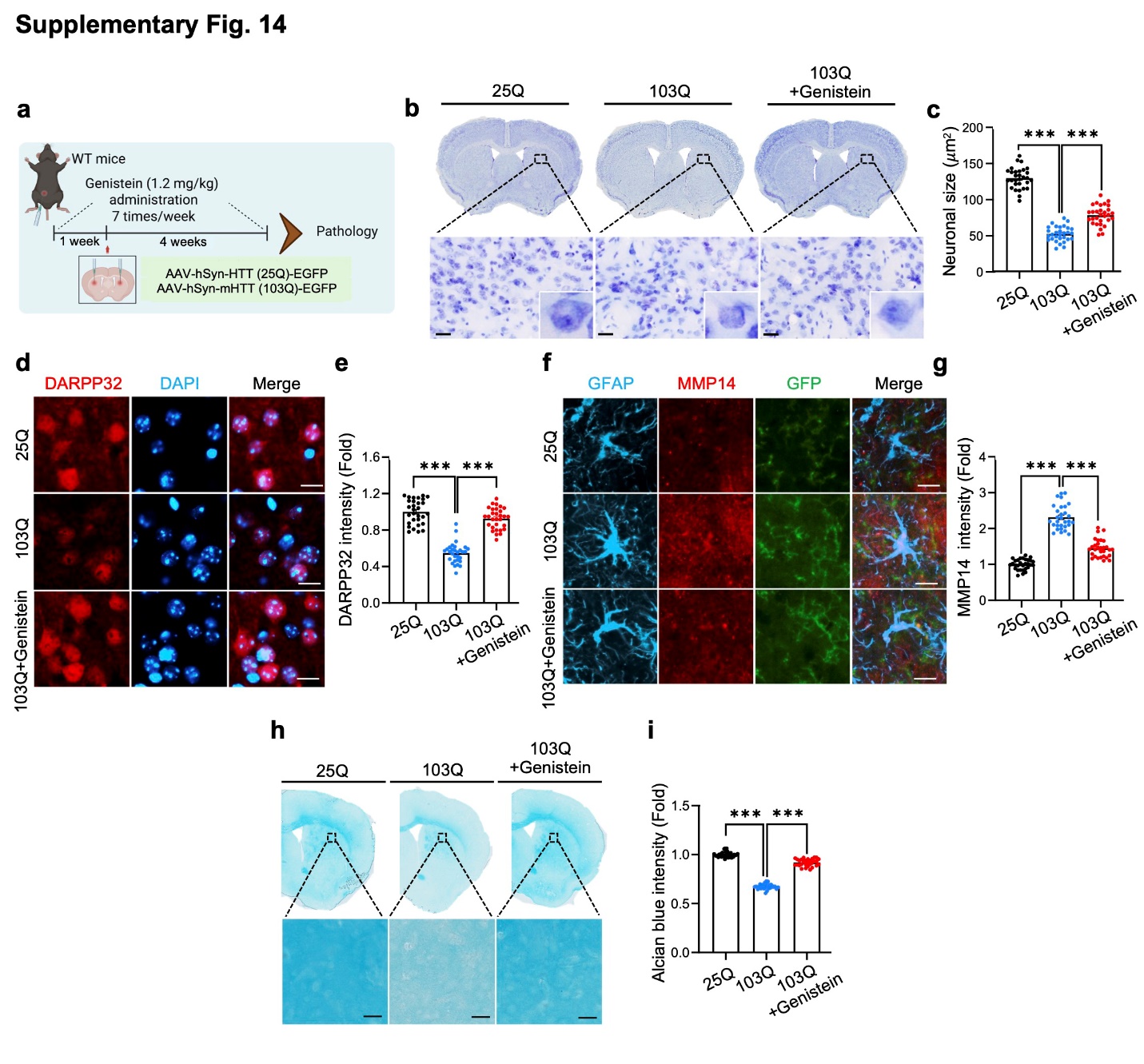
**

Figure. S14. Genistein ameliorates pathology in the AAV-mHTT(103Q) mouse model.

(a) Experimental design for pathological analysis following one month of intraperitoneal administration of genistein or saline in AAV-mHTT(103Q) mice. (b) Representative images of cresyl violet staining in the dorsal striatum. (c) Quantification of neuronal size in three groups of mice (25Q, 103Q, and 103Q + Genistein) showing that genistein preserved neuronal size. A total of 30 cells per group were counted (10 cells per mouse; *N* = 3 mice per group). Error bars indicate mean ± SEM. One-way ANOVA (*, *p* < 0.05; **, *p* < 0.01; ***, *p* < 0.001). (d) Representative images of DARPP-32 immunoreactivity. DARPP-32 expression, which was reduced in 103Q mice, was preserved by genistein. The nuclei were counterstained with DAPI. Scale bars: 20 μm. (e) Quantification of DARPP-32 fluorescence intensity in 25Q, 103Q, and 103Q + Genistein. A total of 30 cells per group were counted (10 cells per mouse; *N* = 3 mice per group). ***, *p* < 0.001. (f) Representative images of MMP14 (red) immunoreactivity in GFAP-positive (blue) astrocytes. Scale bars: 20 μm. (g) Quantification shows that genistein reduced MMP14 immunoreactivity in GFAP-positive cells from 25Q, 103Q, and genistein-treated 103Q groups. A total of 30 cells per group were counted (10 cells per mouse; *N* = 3 mice per group). ***, *p* < 0.001. (h) Representative images of Alcian Blue staining for glycosaminoglycans. Scale bars: 20 μm. (i) Quantification of Alcian Blue density in 25Q, 103Q, and genistein-treated 103Q groups shows that genistein preserved glycosaminoglycan content. Three ROIs (0.05 mm² each) per mouse were analysed (*N* = 3 mice per group). ***, *p* < 0.001.


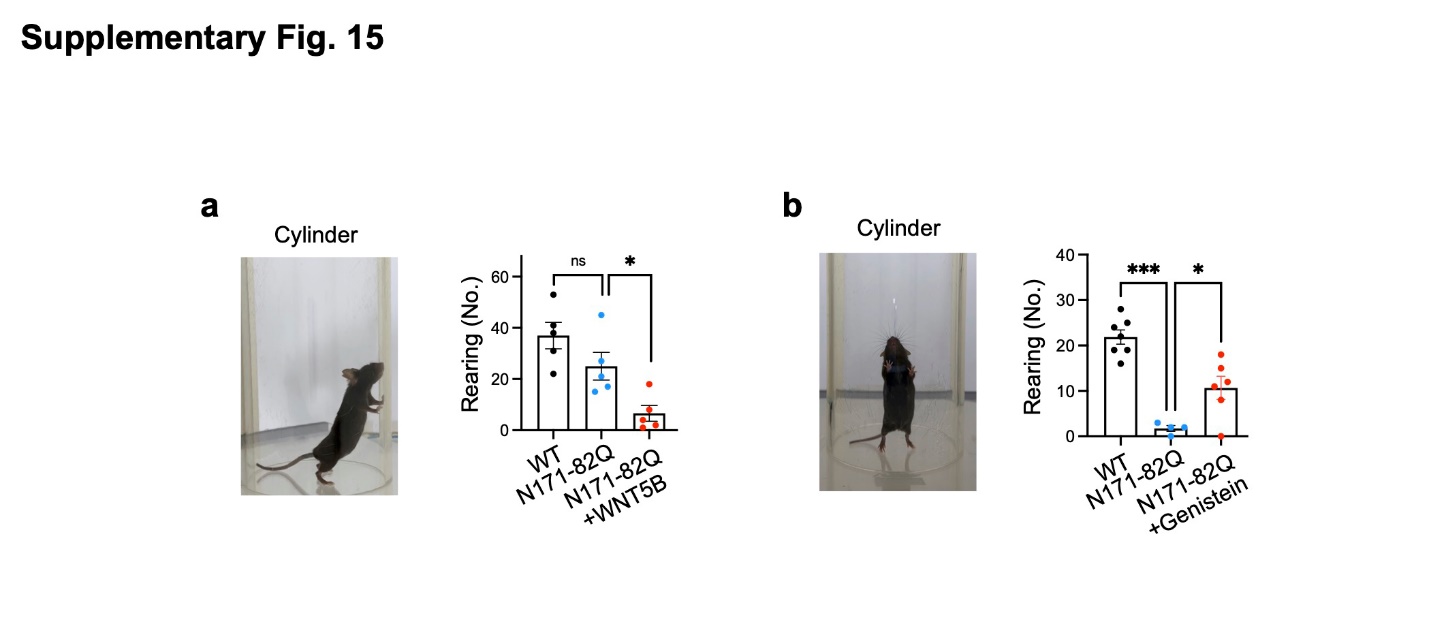


Figure. S15. Cylinder test analysis results of N171-82Q mice under different treatments.

(a) The number of rearings was significantly decreased in N171-82Q + WNT5B mice compared to WT and N171-82Q controls (*N* = 5 mice per group). Error bars indicate mean ± SEM. One-way ANOVA (*, *p* < 0.05; **, *p* < 0.01; ***, *p* < 0.001). (b) Genistein treatment led to a recovery in the number of rearings in N171-82Q mice (WT, *N* = 7; N171-82Q, *N* = 4; N171-82Q + Genistein, *N* = 6). *p* < 0.05; **, *p* < 0.01; ***, *p* < 0.001.

Table. S1. Demographics of normal subjects and HD brain tissue samples

| **Normal** | **Age** | **Gender** | **Disease Grade** | **PMI**  **(Postmortem Interval)**  **Hour** |
| --- | --- | --- | --- | --- |
| 1 | 70 | M | 0 | 12 |
| 2 | 61 | M | 0 | 13 |
| 3 | 63 | M | 0 | 6 |
| 4 | 67 | M | 0 | 10 |
| 5 | 74 | M | 0 | 7 |
| 6 | 56 | M | 0 | 15 |
| **HD** | **Age** | **Gender** | **Disease Grade** | **PMI**  **(Postmortem Interval)**  **Hour** |
| 1 | 71 | M | III | 20 |
| 2 | 43 | M | III | 21 |
| 3 | 68 | M | III | 4 |
| 4 | 69 | M | III | 19 |
| 5 | 75 | M | III | 13 |
| 6 | 53 | M | IV | 16 |

Table. S2. PCR primers that were used for pGL4.14-MMP14 promoter cloning

| **Primer name** | **Sequence (5’→3’)** | **Tm (℃)** |
| --- | --- | --- |
| MMP14(pro-1500)_Acc65I_F | CATATTGGTACCtcctaagattcagatatgtggtc | 53 |
| MMP14(pro-1000)_Acc65I_F | CATATTGGTACCggccaaatgcaattcttg | 53 |
| MMP14(pro-500)_Acc65I_F | CATATTGGTACCgcagcagagggagggaca | 59 |
| MMP14(pro-300)_Acc65I_F | CATATTGGTACCccagaggaatcaagccac | 54 |
| MMP14(pro-200)_Acc65I_F | CATATTGGTACCcaacccctgcagatggcag | 61 |
| MMP14(pro-100)_Acc65I_F | CATATTGGTACCacaaccacgtccccaacc | 60 |
| MMP14(pro)_BglII_R | CATATTAGATCTgttccctctctcgctctc | 56 |

Table. S3. Mice information

| **Figure** | **Group** | **Mouse number** | **Gender** |  | **Group** | **Mouse number** | | **Gender** |
| --- | --- | --- | --- | --- | --- | --- | --- | --- |
| **Fig. 5 and Fig. 6** | **WT-Cont** | 1 | M | **Fig. 8 and Fig. 9** | **WT-Veh** | | 1 | M |
|  |  | 2 | M |  |  |  | 2 | M |
|  |  | 3 | F |  |  |  | 3 | M |
|  |  | 4 | F |  |  |  | 4 | F |
|  |  | 5 | F |  |  |  | 5 | F |
|  |  | 6 | F |  |  |  | 6 | M |
|  |  | 7 | F |  |  |  | 7 | M |
|  | **N171-82Q-Cont** | 1 | M |  | **N171-82Q-Veh** | | 1 | M |
|  |  | 2 | M |  |  |  | 2 | M |
|  |  | 3 | F |  |  |  | 3 | F |
|  |  | 4 | F |  |  |  | 4 | M |
|  |  | 5 | F |  |  |  | 5 | M |
|  |  | 6 | F |  | **N171-82Q-Gen** | | 1 | M |
|  |  | 7 | F |  |  |  | 2 | M |
|  | **N171-82Q+WNT5B** | 1 | M |  |  |  | 3 | M |
|  |  | 2 | M |  |  |  | 4 | F |
|  |  | 3 | F |  |  |  | 5 | F |
|  |  | 4 | F |  |  |  | 6 | F |
|  |  | 5 | F |  |  |  | 7 | F |
|  |  | 6 | F |  |  |  | 8 | M |
|  |  | 7 | F |  | | | | |
| **Fig. 6 and Sup. Fig. 9** | **25Q-Cont** | 1 | M | **Fig. 9 and Sup. Fig. 14** | **25Q-Veh** | | 1 | M |
|  |  | 2 | M |  |  |  | 2 | M |
|  |  | 3 | M |  |  |  | 3 | M |
|  |  | 4 | M |  |  |  | 4 | M |
|  |  | 5 | M |  |  |  | 5 | F |
|  |  | 6 | F |  |  |  | 6 | F |
|  |  | 7 | F |  |  |  | 7 | F |
|  |  | 8 | F |  |  |  | 8 | F |
|  |  | 9 | F |  |  |  | 9 | F |
|  |  | 10 | F |  |  |  | 10 | F |
|  | **103Q-Cont** | 1 | M |  | **103Q-Veh** | | 1 | M |
|  |  | 2 | M |  |  |  | 2 | M |
|  |  | 3 | M |  |  |  | 3 | M |
|  |  | 4 | M |  |  |  | 4 | M |
|  |  | 5 | M |  |  |  | 5 | F |
|  |  | 6 | F |  |  |  | 6 | F |
|  |  | 7 | F |  |  |  | 7 | F |
|  |  | 8 | F |  |  |  | 8 | F |
|  |  | 9 | F |  |  |  | 9 | F |
|  |  | 10 | F |  |  |  | 10 | F |
|  | **103Q+WNT5B** | 1 | M |  | **103Q-Gen** | | 1 | M |
|  |  | 2 | M |  |  |  | 2 | M |
|  |  | 3 | M |  |  |  | 3 | M |
|  |  | 4 | M |  |  |  | 4 | M |
|  |  | 5 | M |  |  |  | 5 | F |
|  |  | 6 | F |  |  |  | 6 | F |
|  |  | 7 | F |  |  |  | 7 | F |
|  |  | 8 | F |  |  |  | 8 | F |
|  |  | 9 | F |  |  |  | 9 | F |
|  |  | 10 | F |  |  |  | 10 | F |
| **Fig. 7** | **25Q-Cont** | 1 | M |  | **103Q-shNfatc2** | | 1 | M |
|  |  | 2 | M |  |  |  | 2 | M |
|  |  | 3 | F |  |  |  | 3 | M |
|  |  | 4 | F |  |  |  | 4 | F |
|  |  | 5 | F |  |  |  | 5 | F |
|  | **103Q-Cont** | 1 | M |  | **103Q-shMmp14** | | 1 | M |
|  |  | 2 | M |  |  |  | 2 | M |
|  |  | 3 | M |  |  |  | 3 | F |
|  |  | 4 | F |  |  |  | 4 | F |
|  |  | 5 | F |  |  |  | 5 | F |
|  | **103Q-shWnt5b** | 1 | M |  | | | | |
|  |  | 2 | M |  |  |  |  |  |
|  |  | 3 | F |  |  |  |  |  |
|  |  | 4 | F |  |  |  |  |  |
|  |  | 5 | F |  |  |  |  |  |

Table. S4. shRNA sequences for knock-down of *Wnt5b*, *Nfatc2*, and *Mmp14*

| **Sequence name** | **Sequence (5’→3’)** |
| --- | --- |
| *Wnt5b* shRNA#1 | TGGCTGATGTCGCCTGCAAAT |
| *Wnt5b* shRNA#2 | GGACAACACATCTGTCTTTGG |
| *Wnt5b* shRNA#3 | GAGAGTGCCAACACCAGTTTC |
| *Nfatc2* shRNA#1 | GCGGCTCCTCTGCCAGCTTCA |
| *Nfatc2* shRNA#2 | GCTTCATTTCTGACACCTTCT |
| *Nfatc2* shRNA#3 | GCAGTTGGTGCCGGCCATTCC |
| *Mmp14* shRNA#1 | GCTGTGGTGTTCCGGATAAGT |
| *Mmp14* shRNA#2 | GGACTGAGATCAAGGCCAATG |
| *Mmp14* shRNA#3 | GGCCTCAAGTGGCAGCATAAT |

**Supplementary References**

1. Matys, V. et al. TRANSFAC and its module TRANSCompel: transcriptional gene regulation in eukaryotes. Nucleic Acids Res. 34, D108–D110 (2006).
2. Yao, Z. et al. A high-resolution transcriptomic and spatial atlas of cell types in the whole mouse brain. Nature 624, 317–332 (2023).
